# Fine-scale genetic structure in the critically endangered red-fronted macaw in the absence of geographic and ecological barriers

**DOI:** 10.1038/s41598-020-79575-6

**Published:** 2021-01-12

**Authors:** Guillermo Blanco, Francisco Morinha, Séverine Roques, Fernando Hiraldo, Abraham Rojas, José L. Tella

**Affiliations:** 1 Department of Evolutionary Ecology, National Museum of Natural Sciences (MNCN), Spanish National Research Council (CSIC), José Gutiérrez Abascal 2, 28006 Madrid, Spain; 2 Irstea-UR EABX, Ecosystèmes Aquatiques et Changements Globaux, Equipe FREEMA, 50 avenue de Verdun, 33 612 Cestas, France; 3 Department of Conservation Biology, Estación Biológica de Doñana (CSIC), Avda. Américo Vespucio, 41092 Sevilla, Spain; 4 Zoológico Municipal de Fauna Sudamericana, Radial 27, Tercer Anillo Interno, Santa Cruz de la Sierra, Bolivia

**Keywords:** Conservation biology, Molecular ecology

## Abstract

Behavioural and socio-cultural traits are recognized in the restriction of gene flow in species with high cognitive capacity and complex societies. This isolation by social barriers has been generally overlooked in threatened species by assuming disrupted gene flow due to population fragmentation and decline. We examine the genetic structure and ecology of the global population of the Critically Endangered red-fronted macaw (*Ara rubrogenys*), an endemic species to the inter-Andean valleys of Bolivia. We found a fine-scale genetic structuring in four genetic clusters. Genetic diversity was higher in wild compared to captive-bred macaws, but similar to that of captive wild-caught macaws. We found no clear evidence of severe genetic erosion in the population in recent decades, but it was patent in historic times, overlapping with drastic human habitat transformation and macaw persecution over millennia. We found no evidence of geographical and ecological barriers, owing to the high dispersal ability, nesting and foraging habits between genetic clusters. The lack of genetic intermixing despite long-distance foraging and seasonal movements suggests recruitment in natal colonies and other social factors reinforcing philopatry-related genetic structure. Conservation efforts should be specifically focussed on major threats in each genetic cluster as independent conservation units, and also considered in ex-situ management.

## Introduction

Genetic structure of populations is the result of multiple ecological and evolutionary forces acting in concert^1,2^. Limitations to gene flow by geographic barriers or limited dispersal ability are often highlighted as primary causes of differentiation and genetic structure owing to distance among population nuclei^3^. This process is often linked to geo-climatic features, which can also be a main source of ecological filters to gene flow leading to isolation by adaptation^1,4^. Distinguishing between these major mechanisms by their contribution to genetic structure of populations is often challenging for terrestrial birds with high dispersal capacity and generalised habitat use^5^. Recently, behaviour and socio-cultural traits have become increasingly recognized in the restriction of gene flow among populations of species with high cognitive capacity and complex societies, especially mammals like cetaceans and primates including humans^6^.

In birds, evidence of isolation by social barriers is still scarce and restricted to species with complex societies^7,8^. Crows, jays and other corvids (Corvidae, Passeriformes) and parrots and allies (Psittaciformes), are among the birds with a recognised high cognitive capacity, evolving in parallel to complex patterns of social behaviour driving population organisation^9,10^. Social barriers to gene flow can act through recognition among individuals from population nuclei showing particular social and behavioural identities^11^. For instance, socio-cultural factors may have played a role in driving genetic structure associated with tool use even at small spatial scales^12,13^. Complex hierarchical, social and ritualised interactions among group members, high mate and site fidelity, and complex vocalisations have been suggested to be involved in the extreme genetic structure in the Red-billed chough (*Pyrrhocorax pyrrhocorax*) despite high dispersal ability and large population size^8^. Evidence of these social traits and population organisation patterns also exists for parrots^14,15^, especially linked to complex vocalisations and dialects^16,17^. However, the contribution of geographical, ecological and social barriers to genetic structure of parrot populations has been poorly investigated at large population scales.

Evaluating the processes and mechanisms that determine the genetic structure of populations is essential to understand their demographic and ecological dynamics^18^. In general, emigration and immigration between breeding nuclei have been highlighted as key factors driving the genetic structure of populations, since even a small number of these exchanges per generation can have a great influence on population homogenisation due to the mixture of different genetic pools^19^. Depending on the magnitude of connectivity between populations and on the reproductive outcomes of dispersers, the genetic structure and diversity can be more or less pronounced. This variability can be further modulated by historical trends due to past and ongoing threats eroding the viability of isolated nuclei and of highly fragmented and small populations of threatened species^20–22^. The influence of isolation by social barriers on the genetic structure of threatened species has been generally overlooked, often through assumptions that connectivity is generally disrupted due to population fragmentation associated with habitat loss and population declines. However, fine-scale genetic structure can be an intrinsic feature evolving in particular species^23,24^, as promoted by socio-cultural forces driving pairing, dispersal and group cohesion even in large and widely distributed populations of social species^8,25^. A comprehensive evaluation of these traits is paramount to understanding the structure and functioning of metapopulations and for adequate conservation management, including captive breeding and reintroduction of threatened species^26,27^.

Here, we examine the global population genetic structure of the Critically Endangered red-fronted macaw (*Ara rubrogenys*). This species represents a suitable study model to investigate the potential drivers of genetic structuration of rare social species distributed in discrete population nuclei associated with breeding colonies interconnected by long-distance movements^28–30^. We inferred population structure from individual genotypes at nine microsatellite loci from a large part of the main breeding areas throughout its restricted global distribution range in the inter-Andean valleys of Bolivia^30^. We focussed on genetic diversity, inbreeding, differentiation and connectivity among population nuclei, and tested for patterns of contemporary demography through bottleneck analysis. Our main goal is to explore potential mechanisms promoting and limiting gene flow between subpopulations and genetic clusters, and whether isolating processes related to geographic barriers to dispersal, adaptation to particular habitats, and social organisation can explain the current genetic structure of the global population. We also evaluated the genetic characteristics of several captive populations to assess their most likely wild population nuclei of origin. We discuss ways to apply the information about genetic structure to the conservation of the whole population or of particular genetic clusters, and how this should drive ex-situ conservation management, including captive breeding and reintroduction.

## Results

### Sample inclusion criteria and population genetic structure

In total, 162 alleles were amplified to estimate error rates, i.e. 9 samples typed for 9 alleles, but only 104 (64%) were considered in the analysis after excluding alleles that did not amplify in at least one of the reactions. These unsuccessful amplifications are related to low DNA quantities obtained from feather samples. No evidence of false alleles were detected and only three allelic dropouts (one for locus AgGT90 and two for locus Peeμ11) were identified. The overall error rate was low (2.9%) considering that low quality DNA samples are more prone to genotype errors. The cumulative probabilities estimated for the nine microsatellite loci were *P*_(ID)_ < 0.0001 and *P*_(ID)sib_ = 0.0013 (Table S1, Fig. S1).

The analysis of genetic relatedness and/or molecular sexing identified 94 different wild individuals (genotypes of each individual are shown in Appendix S1). Among them, a total of twelve full-sibs were identified in different population groups (three in W1; one in W2; six in W3; two in W4). The analysis of genetic differentiation with and without full-sibs did not affect the results of the clustering analysis. Thus, the full data set with 94 wild individuals was used in all subsequent analyses. The complete data set includes 146 individuals (94 from wild populations and 52 from captive birds) with low levels of missing data (one individual missing three loci; eight individuals missing two loci; 18 individuals missing one locus) (Appendix S1).

**Figure 1:**
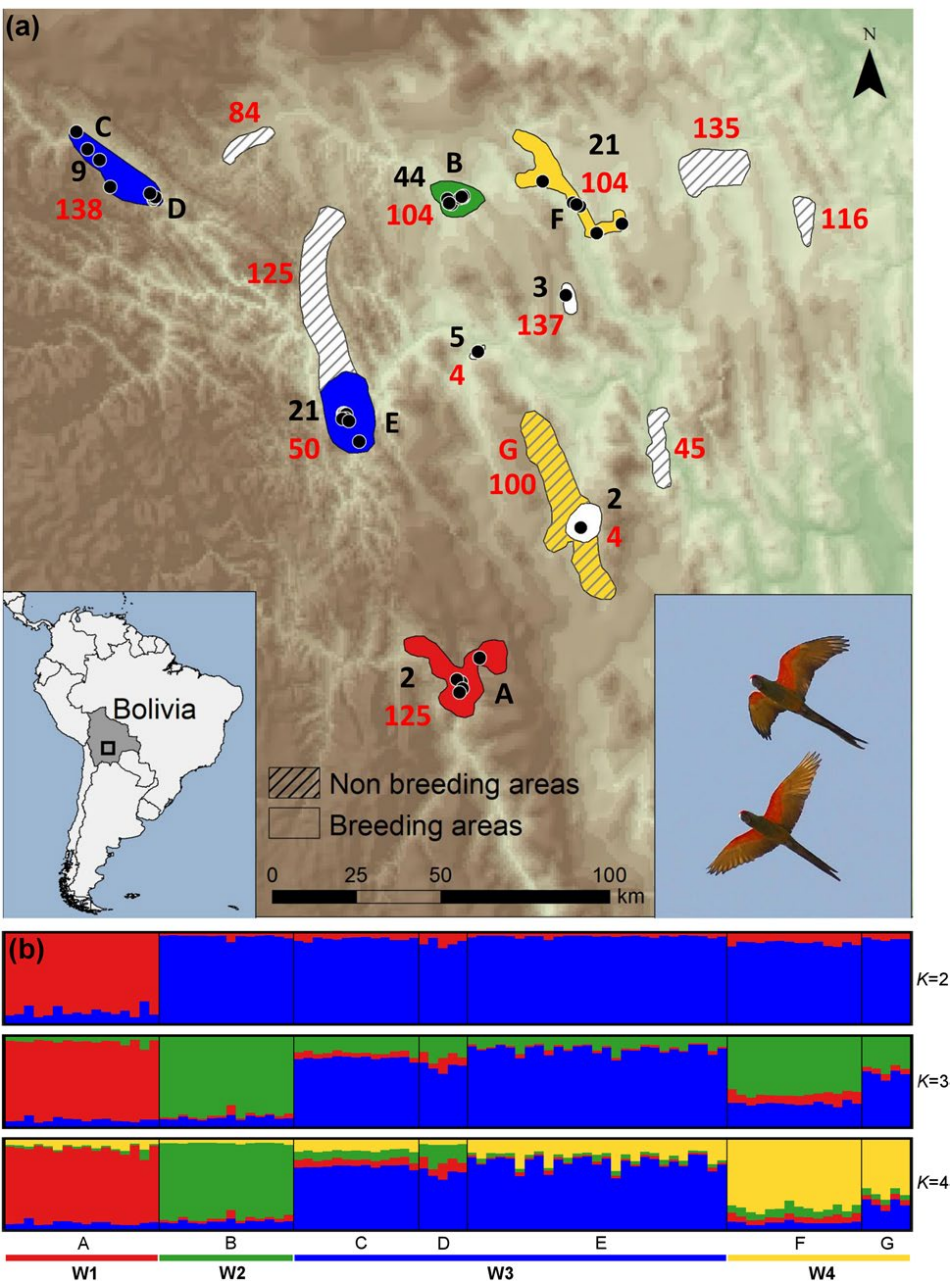
(**a**) Distribution range of the red-fronted macaw showing the sampled population nuclei (represented by letter codes: black letters for breeding nuclei, red letter for the single non-breeding area sampled) pooled in genetic clusters (represented by colours). The number of breeding pairs in each population nucleus at the time this study was conducted is represented by black numbers, while the maximum flock size in each breeding and non-breeding areas is represented by red numbers. Black points represent breeding colonies. (**b**) Distinct genetic clusters from the Bayesian clustering analysis with prior information on sampling location are shown for several *K* clusters. Each vertical line corresponds to one individual and the different colours represent the assignment to each cluster, with *K* = 4 as the optimal number of clusters estimated. Map was generated with ArcGIS 10.5 software (ESRI, Redland, USA, https://desktop.arcgis.com/en/) and modified with Microsoft PowerPoint 2010 (Microsoft Corporation, Redmond, WA, USA, https://www.microsoft.com/pt-pt/microsoft-365/previous-versions/office-2010), both software under CSIC Organizational License. Background was generated from Digital Elevation Model available from USGS (Global 30 Arc-Second Elevation, GTOPO30), freely available at https://www.usgs.gov/centers/eros/science/usgs-eros-archive-digital-elevation-global-30-arc-second-elevation-gtopo30?qt-science_center_objects=0#qt-science_center_objects. Author of the photograph: José L. Tella.

The Bayesian clustering analysis of multilocus genotypes using sample location prior clearly revealed a small-scale genetic structure of four population nuclei, namely W1 (A), W2 (B), W3 (C, D, E) and W4 (F, G) (Fig. 1a,b). The number of clusters was supported by both approaches used to estimate the optimal *K* value (Fig. 1b; Fig. S2a, b). The hierarchical clustering performed in the second level of STRUCTURE did not detect evidence of population differentiation between sites of clusters W3 and W4. No genetic differentiation was detected in the analysis without sample location priors. All pairwise *F*_st_ and *altitude* estimates were significant (P < 0.01) and highly correlated (*r* = 0.99; *P* < 0.05) (Table 1). The highest values involved the comparisons between cluster W1 and the other clusters. The lowest values involved adjacent clusters, W2-W4 and W3-W4 respectively. A PCoA analysis among wild population groups also supports the STRUCTURE, *F*_st_ and *D*_est_ estimates (Supplementary Fig. S3a, b).

**Table 1:** Pairwise values of *F*_*st*_ (below diagonal) and *D*_est_ (above diagonal) among genetic clusters of red-fronted macaw in the wild.

	Genetic cluster			
	W1	W2	W3	W4
W1	–	0.157	0.086	0.114
W2	0.106	–	0.083	0.058
W3	0.059	0.049	–	0.055
W4	0.074	0.030	0.036	–

All estimated values are significant (*P* < 0.01).

**Figure 2:**
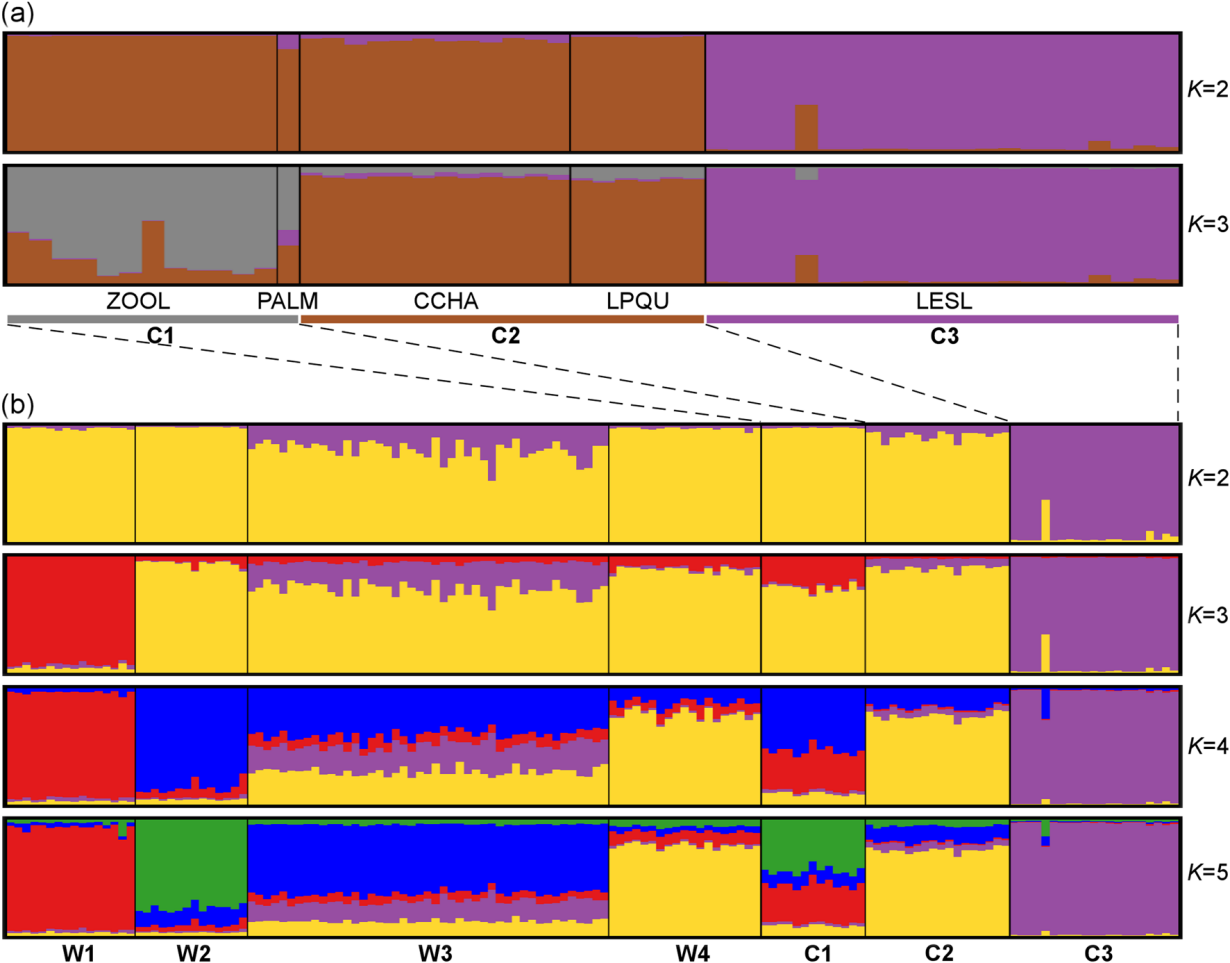
(**a**) Distinct genetic clusters from the Bayesian clustering analysis with prior information on sampling location are shown for several *K* clusters for captive red-fronted macaws, where the optimal number of clusters was estimated for *K* = 3. (**b**) Clustering analysis combining captive and wild individuals, where the optimal number of clusters was estimated for *K* = 5. Each vertical line corresponds to one individual and the different colours represent the assignment to each cluster. Colours assigned to each cluster correspond to the genetic clusters obtained for the wild population (see Fig. 1), except for the cluster C3 that cannot be assigned to any cluster in the wild.

The captive individuals were clustered into three distinct genetic groups, namely C1 (ZOOL and PALM), C2 (CCHA and LPQU) and C3 (LESL) (Fig. 2a). The Bayesian clustering analysis of the dataset including both captive and wild individuals resulted in five different genetic clusters (Fig. 2b). The W4 wild population and C2 captive group belong to the same cluster. The C1 captive group showed a mixed ancestry from individuals belonging to W1 and W2 wild populations (Fig. 2b). These cluster patterns were obtained using the LOCPRIOR option. No genetic structure was observed in both analyses without sample location priors. The optimal *K* values were estimated using the previous developed approach for complex and highly structured data sets. The *∆K* method underestimate the number of clusters in the first level of structure analysis when captive groups where included (*K* = 2 for both clustering analysis, Fig. S2c–f).

PCoA including both captive and wild individuals also indicated that the genetic composition of those groups is close, with the exception of the C3 group, which is genetically differentiated from the rest (Supplementary Fig. S3c, d).

### Genetic diversity and demographic events

The polymorphic microsatellite loci selected for this study (Table S2) allowed the amplification of 62 different alleles, ranging from 3 (MmGT057 and UnaCT74) to 13 (AgGT21), with an average of 6.89 alleles. At a locus level, average values of allelic richness ranged between 2.04 (UnaCT74) and 6.56 (AgGT21), mean observed heterozygosity ranged between 0.25 (UnaCT74) and 0.81 (UnaCT43), and the mean expected heterozygosity ranged between 0.23 (UnaCT74) and 0.74 (UnaCT43 and AgGT90) (Supplementary Table S3). The power of discrimination of most markers is supported by PIC values (global mean of 0.52 ± 0.22), although low PIC values were obtained for MmGT057 (0.25) and UnaCT74 (0.20). Average *F*_*IS*_ values ranged between − 0.08 (UnaCT43 and UnaCT74) and 0.07 (AgGT90). Deviation from Hardy–Weinberg equilibrium (HWE) after Bonferroni correction was detected only for locus (AgGT90) in the W4 cluster (Supplementary Table S3). No significant evidence for linkage disequilibrium was found, suggesting that the assayed loci assorted independently.

At a population level, no evidence of deviations from Hardy–Weinberg equilibrium (HWE) was detected in the wild population, except for W1, which showed a significant heterozygote deficit (Table 2). This can be a result biased by the substantially lower *H*_*O*_ than *H*_*E*_ of the locus Peeμ11 in cluster W1 (Table S3), probably influenced by allelic dropout and some missing data at this locus. *F*_*IS*_ for the cluster W1 (0.12) was the largest of all the wild groups (Table 2). The presence of null alleles is unlikely given the strict selection of markers to discard such bias. Captive groups were in HW equilibrium, except C3, which showed significant heterozygote excess (Table 2) suggesting that breeding between individuals did not occur by chance.

**Table 2:** Genetic diversity for each cluster of wild and captive red-fronted macaw.

Genetic cluster (code)	*n*	*N*_*A*_	*A*_*R*_	*H*_*O*_	*H*_*E*_	HW P value (Heterozyg. Deficit)	HW P value (Heterozyg. Excess)	*F*_*IS*_
W1 (A)	16	4.44 (2.07)	4.17 (2.00)	0.46 (0.26)	0.50 (0.30)	< 0.01**	0.99	0.12*
W2 (B)	14	4.44 (2.07)	4.18 (1.83)	0.56 (0.20)	0.58 (0.18)	0.09	0.91	0.07
W3 (C, D, E)	45	5.78 (2.68)	4.47 (1.78)	0.59 (0.26)	0.59 (0.24)	0.29	0.71	0.01
W4 (F, G)	19	5.00 (2.18)	4.60 (1.98)	0.61 (0.18)	0.63 (0.18)	0.05	0.95	0.07
C1 (ZOOL, PALM)	13	4.22 (1.56)	4.05 (1.42)	0.61 (0.23)	0.54 (0.19)	0.89	0.11	-0.09
C2 (CCHA, LPQU)	18	5.11 (3.10)	4.67 (2.46)	0.65 (0.29)	0.61 (0.23)	0.88	0.12	-0.05
C3 (LESL)	21	3.22 (1.39)	2.92 (1.10)	0.63 (0.28)	0.49 (0.21)	1.00	< 0.01**	-0.27

Codes represent the sampling sites as shown in Table 3.

*n* number of samples, *N*_*A*_ number of alleles, *A*_*R*_ allelic richness, *H*_*O*_ observed heterozygosity, *H*_*E*_ expected heterozygosity, *F*_*IS*_ inbreeding coefficients.

*p < 0.05; **p < 0.01.

A mean of 4.25 alleles per locus was detected among the 94 genotyped wild macaws. We found a similar value (*N*_*A*_ = 4.50) in captive samples from Bolivia, while in captive-bred individuals from Spain the diversity was extremely low (*N*_*A*_ = 2.7) (Table 3). Mean observed and expected heterozygosity in wild population clusters were 0.56 and 0.58, respectively. The heterozygosity value was the lowest in W1 (*H*_*O*_ = 0.46) (Table 2). Mean diversity values of captive groups C1 and C2 were similar to those found in the wild populations, but lower diversity indices were found in C3 (Table 2). In captive clusters, mean values of observed and expected heterozygosity were 0.63 and 0.55, respectively. In all captive groups, *H*_*O*_ was higher than *H*_*E*_, what is expected after a recent reduction in size and/or founder event. The magnitude of allelic richness values was similar across wild and captive genetic clusters, except C3, which showed reduced values of allelic richness (2.92 ± 1.10) and number of alleles (3.22 ± 1.39) (Table 2). Only six private alleles were found in both wild (W1 and W2) and captive (C2 and C3) genetic clusters (Table S4).

**Table 3:** Sampling characterisation, circumstances around sample collection and microsatellite genetic diversity indices for all localities.

Country	Area (code)	Origin	Sample type	*n*^a^	Sample collection	***N***_***A***_	***H***_***O***_	***H***_***E***_
Bolivia	Icla (A)	Wild	Feather	16	Beneath the trees used as main communal roost	4.44 (2.07)	0.46 (0.26)	0.50 (0.30)
Bolivia	Omereque (B)	Wild	Feather	14	Beneath the cliffs with breeding colonies	4.44 (2.07)	0.56 (0.20)	0.58 (0.18)
Bolivia	Río Caine (C)	Wild	Feather	13	Foraging areas of pairs with offspring close to the breeding colonies	4.33 (2.06)	0.57 (0.33)	0.54 (0.26)
Bolivia	Torotoro (D)	Wild	Blood	5	Local nestlings poached from a particular locality (Torotoro)	3.33 (1.32)	0.51 (0.39)	0.50 (0.26)
Bolivia	Río Chico (E)	Wild	Feather	27	Beneath the cliffs with breeding colonies	4.89 (2.15)	0.61 (0.24)	0.59 (0.21)
Bolivia	Anamal (F)	Wild	Feather	14	Beneath the cliffs with breeding colonies	4.78 (1.99)	0.60 (0.16)	0.63 (0.17)
Bolivia	Tomina (G)	Wild	Blood	5	Adults kept as pets, illegally trapped in foraging areas far from the breeding colonies	3.56 (1.88)	0.62 (0.34)	0.56 (0.21)
Bolivia	Sta. Cruz Zoo (ZOOL)	Captive	Blood	12	Zoological Park of Santa Cruz de la Sierra; Exposed to the public, not breeding; Unknown wild origin from poaching	4.00 (1.41)	0.62 (0.22)	0.54 (0.19)
Bolivia	Cochabamba (CCHA)	Captive	Blood	12	Private owner in Cochabamba; Mostly poached nestlings, not reproducing in captivity	1.44 (0.53)	0.44 (0.53)	0.22 (0.26)
Spain	Loro Parque (LPQU)	Captive	Blood	6	Individuals legally purchased at Loro Parque, a zoological park in Santa Cruz de Tenerife, Canary Islands	5.00 (2.83)	0.62 (0.26)	0.61 (0.22)
Spain	Palmitos Park (PALM)	Captive	Blood	1	Individual legally purchased at Palmitos Park, a zoological park in Gran Canaria, Canary Islands	3.44 (1.33)	0.72 (0.38)	0.54 (0.28)
Spain	Málaga (LESL)	Captive	Feather/blood	21	Private collection breeding in captivity; Originated from six wild macaws internationally traded and legally purchased 30 years ago in England	3.22 (1.39)	0.63 (0.28)	0.49 (0.21)

*N*_*A*_ number of alleles, *H*_*O*_ observed heterozygosity, *H*_*E*_ expected heterozygosity.

^a^Number of samples included in the final data set considering only one sample per individual and the missing data threshold.

The results of the analysis with BOTTLENECK suggested historical population contraction events in populations W3 and W4 only when assuming an infinite allele model (IAM; Table 4). No evidence of bottlenecks was found using the two-phase model (TPM), stepwise-mutation model (SMM) or the mode-shift test, although a significant value for heterozygote deficiency was obtained for population W3 using TPM (Table 4). The M-ratio test consistently suggests population contractions (values of M < Mc) in all clusters considering all pre-bottleneck *N*_*e*_ values (Table 4).

**Table 4:** Results of the bottleneck analysis.

Bottleneck				Mode-shift	*M-ratio* (M_c_)			
Genetic cluster	TPM *P*-values	SMM *P*-values	IAM *P*-values		M	*θ* = 0.1	*θ* = 1	*θ* = 10
W1	0.180	0.180	0.410	Normal L-shaped	0.421	**0.763**	**0.693**	**0.590**
W2	0.545	0.633	0.082	Normal L-shaped	0.528	**0.767**	**0.695**	**0.575**
W3	0.936	0.986*	**0.009**	Normal L-shaped	0.265	**0.767**	**0.706**	**0.669**
W4	0.248	0.500	**0.005**	Normal L-shaped	0.272	**0.761**	**0.698**	**0.605**

*P*-values of the one-tail Wilcoxon test for heterozygote excess are shown for the Two-Phase Model (TPM), Stepwise Mutation Model (SMM) and Infinite Allele Model (IAM). For the Mode-shift test, modes obtained for each population are indicated. Values of the observed M-ratio (M) and critical ratio (Mc) were estimated for three values of pre-bottleneck *θ* that correspond to *N*_*e*_ values of 50, 500 and 5000, respectively. Values in bold suggest historic bottleneck events.

*Significant *p*-values were obtained for heterozygote deficiency (*p* < 0.05).

### Migration rates, relatedness and gene flow

Migration rates (*m*) among genetic clusters were generally low, with a global mean value of 0.056 (ranging from 0.016 to 0.192) (Table 5, Supplementary Fig. S4). Moderate values were obtained only for population W3, with a mean value of 0.130 ± 0.063. Assignment tests implemented in GENECLASS2 identified only 3 potential migrants with a *p*-value < 0.01. One migrant from population W2 was found in populations W1 and W4, and one migrant from W4 was found in W2.

**Table 5:** Contemporary gene flow (*m*, migration rate, results from BAYESASS) estimated between the four wild genetic clusters of the red-fronted macaw in the wild.

Population	To			
From	W1	W2	W3	W4
W1	0.8812 (0.0477)	0.0248 (0.0251)	0.0654 (0.0394)	0.0286(0.0280)
W2	0.0314 (0.0290)	0.7293 (0.0542)	0.1920 (0.0491)	0.0473(0.0413)
W3	0.0348 (0.0228)	0.0155 (0.0155)	0.9281 (0.0274)	0.0216(0.0173)
W4	0.0288 (0.0270)	0.0495 (0.0509)	0.1311 (0.0446)	0.7907(0.0565)

Standard deviation of the marginal posterior distribution for each estimate is noted in parentheses. Migration rates indicate the fraction of individuals in the population of destination that are migrants derived from the source population (per generation).

Results from the 2MOD analysis in wild macaws supported a gene flow-drift equilibrium model over a drift-alone model [p (gene flow) = 0.969]. The cluster W1 showed the largest identity by descent value (*F*-value = 0.104) under this model and the lowest estimates of immigrants per generation (*M* = 4.10), which can be inferred as the product of drift given its higher geographic isolation. Different *F*-values and immigrants per generation were estimated for the other three populations, namely W2 (*F*-value = 0.090; *M* = 5.06), W3 (*F*-value = 0.034; *M* = 13.38) and W4 (*F*-value = 0.048; *M* = 9.92).

The cluster W1 showed the highest relatedness values (0.25 ± 0.24) among wild samples (Supplementary Fig. S5, Appendix S2). The highest values of *r* in captive groups were obtained for C1 (0.17 ± 0.25) and C3 (0.39 ± 0.22). The remaining wild and captive groups have mean r values ranging from -0.03 ± 0.24 (W4) to 0.10 ± 0.24 (W2) (Supplementary Fig. S5, Appendix S2). Estimates of effective population size (*N*_*e*_) for each population group were 57 (CI = 14-inf) for W1, 105 (CI = 17-inf) for W2, 81 (CI = 35–2698) for W3 and 38 (CI = 16-inf) for W4. The value of *N*_*e*_ considering all individuals as a single population was 87 (CI = 52–188).

The analysis of IBD showed a tendency of lower genetic differences at higher geographic proximity, although the correlation between genetic and geographic distances was not significant (Mantel test; Z = 109.12, r = 0.55, P = 0.15). The RMA regression line for *F*_*st*_ explained 28.14% of the variation (Supplementary Fig. S6a). The results were also not significant when the population further south (A-W1) was excluded from the analysis (Mantel test; Z = 42.19, r = 0.08, P = 0.86), where the RMA explained only 3.6% of the variation (Supplementary Fig. S6b).

### Breeding colonies, flock size and diet

Overall, we recorded the characteristics of the 42 breeding colonies located within the four genetic clusters (Supplementary Table S5), plus one colony from a population nuclei not sampled for genetic analyses. All sampled colonies were located on rocky cliffs of variable size and orientation along the main rivers at elevations ranging from 1300 to 2600 m a.s.l. (Supplementary Table S5). Univariate comparisons showed that elevation of the breeding colonies differed between genetic clusters, with colonies within genetic cluster W1 located at the highest elevations and those in the genetic cluster W4 at the lowest elevations (Supplementary Table S5). Length and width of the nesting cliffs, their orientation and distance to the nearest river did not differ between genetic clusters (Supplementary Table S5).

Maximum flock size in each breeding and non-breeding area was higher than the number of breeding individuals in each breeding area (Fig. 1a). Non-breeding areas were located at variable distances from the nearest breeding areas, and often at intermediate distances between breeding areas corresponding to different genetic clusters. Maximum flock size in non-breeding areas were also generally higher than those in the nearest breeding area and genetic cluster (Fig. 1a).

The foraging activity of 197 macaw flocks comprising 2180 individuals was recorded in the breeding areas, while 23 foraging flocks comprising 361 macaws were observed in the non-breeding areas (Table 6). The size of foraging flocks in the breeding areas did not differ between genetic clusters (Kruskal–Wallis test, H = 3.69, df = 3, P = 0.29, n = 177), or between breeding areas (mean ± SD = 11.1 ± 17.0, n = 197, pooling all genetic clusters plus the breeding area not sampled for genetic samples) and non-breeding areas (15.7 ± 17.8, n = 23) (Kruskal–Wallis test, H = 3.36, df = 1, P = 0.07, n = 220).

**Table 6:** Diet composition of the red-fronted macaw in each genetic cluster, and pooled breeding and non-breeding areas, according to flocks (F) and individuals (I).

Species	Genetic cluster				Breeding areas		Non-breeding areas	
	W1	W2	W3	W4	Total		Total	
	F/I	F/I	F/I	F/I	F (%)	I (%)	F (%)	I (%)
*Jatropha hieronymi*	4/14	21/139	5/36	4/18	45 (22.8)	402 (18.4)	2 (8.7)	12 (3.3)
*Schinopsis marginata*	2/8	5/17	14/166	23/260	44 (22.3)	451 (20.7)	5 (21.7)	42 (11.6)
***Zea mays***	1/2	10/69	10/213	6/212	27 (13.7)	496 (22.8)	10 (43.5)	246 (68.1)
*Prosopis kuntzei*		3/30	17/69		20 (10.2)	99 (4.5)		
***Arachis hypogaea***	4/92	1/4	8/145		13 (6.6)	241 (11.1)	5 (21.7)	59 (16.3)
*Prosopis alba*			7/139	2/5	9 (4.6)	144 (6.6)		
*Ziziphus mistol*				1/24	9 (4.6)	84 (3.9)		
*Schinus molle*	1/1	1/4	6/78		8 (4.1)	83 (3.8)		
*Anadenanthera colubrina*		1/6		4/17	5 (2.5)	23 (1.1)		
*Loxopterygium grisebachii*		2/11		2/12	4 (2.0)	23 (1.1)		
*Cnidoscolus* sp*.*			3/11		4 (2.0)	23 (1.1)		
*Cenchrus* sp*.*		2/13			2 (1.0)	13 (0.6)		
*Neoraimondia herzogiana*		1/11		1/2	2 (1.0)	13 (0.6)		
*Anisocapparis speciosa*			1/22		1 (0.5)	22 (1.0)		
*Aspidosperma quebracho-blanco*							1 (4.3)	2 (0.6)
*Celtis ehrenbergiana*				1/6	1 (0.5)	6 (0.3)		
*Parkinsonia praecox*		1/3			1 (0.5)	3 (0.1)		
*Selaginella sellowii*		1/42			1 (0.5)	42 (1.9)		
*Senegalia visco*				1/12	1 (0.5)	12 (0.6)		
Total	12/117	49/349	71/879	45/568	197 (100)	2180 (100)	23 (100)	361 (100)
*n* plant species	5	12	9	10	18		5	

Cultivated species are shown in bold.

A total of 19 plant species were recorded being consumed by red-fronted macaws (Table 6). Diet composition showed no clear differences among genetic clusters, mainly because three vegetal species, namely Palo Borracho (*Jatropha hieronymi*, Euphorbiaceae), Soto (*Schinopsis marginata*, Anacardiaceae) and cultivated maize (*Zea mays*, Poaceae), were dominantly exploited by flocks and individuals in all areas (Table 6). Peanut (*Arachis hypogaea*) crops were also exploited frequently in all clusters (especially in W1) except cluster W4. The exploitation of these four vegetal species accounts for about 52–92% of foraging flocks and 64–99% of foraging individuals in the areas corresponding to each genetic cluster. All the remaining vegetal species sum > 10.2% of flocks or > 6.6% of foraging individuals in the breeding areas. Foraging flocks outside the breeding areas were recorded feeding on the same four plant species, although the sample size was lower than in the breeding areas (Table 6). One species, Quebracho Blanco (*Aspidosperma quebracho-blanco*, Apocynaceae), was infrequently consumed in the non-breeding areas and not consumed in the breeding areas. The exploitation of crops was lower in the breeding areas (20.3% of flocks and 33.8% of individuals) than in the non-breeding areas (65.2% of flocks and 84.5% of individuals) (Fisher exact test, both P < 0.0001). The size of flocks exploiting crops was similar between breeding and non-breeding areas (Mann–Whitney U-test, z = 1.09, P = 0.27). Pooling breeding and non-breeding areas and plant species, the size of flocks feeding on crops (mean ± SD = 18.9 ± 24.9, n = 55) was larger than that of flocks feeding on non-cultivated plants (9.1 ± 12.8, n = 165) (z = 2.96, P = 0.003).

## Discussion

### Genetic structure and diversity

In this study, we found evidence for a fine-scale genetic structuring across the whole population of the red-fronted macaw. Four genetic clusters were defined, corresponding to different breeding colonies spatially grouped along main rivers. The genetic structure over a much smaller spatial scale than that observed for other large parrot species with high dispersal ability^31–33^ is unexpected given the short distances between population nuclei and the great dispersal potential of the red-fronted macaw^28–30^.

Our genetic assay successfully identified a total of 94 wild individuals, which represent about 10% of the extant world population estimated in the last published survey^30^, when our genetic survey was also conducted. Despite the successful cross-species transfer of the nine microsatellites markers selected for this study, we cannot exclude a potential effect of ascertainment bias (i.e. microsatellite repeats tend to be longer and more polymorphic in the source species than in the new target species)^34^. Therefore, the comparison of our microsatellite data with results obtained for other species should be done cautiously. PIC values indicated a good informative level of these microsatellites, including five highly informative (PIC > 0.5), three reasonably informative (0.5 > PIC > 0.25) and only one slightly informative (PIC < 0.25). The cumulative probability of identity of our microsatellite data set was less than 0.01, as recommended for population studies using noninvasive sampling^35^. The collection of moulted feathers in the colonies and their close surroundings was relatively precise in assigning individuals to their actual population nuclei of breeding and natal origin, although this could only be ensured by sampling nestlings in their colonies. While the genetic heterogeneity of the whole population may reflect biologically relevant differences between population nuclei, low gene exchanges among sampling sites were also detected, as shown by clustering analysis, mean assignment values and estimates of migration rates. Therefore, the apparently low gene flow among genetic clusters may simply indicate the inclusion in the analysis of a few transient individuals moving from adjacent colonies, rather than actual genetic intermixing across generations that would have resulted in a lack of genetic structure^19,36^. The genetic clusters grouped nearby breeding colonies across geographically continuous cliffs along major water courses, although one of the clusters (W3) included discontinuous breeding areas separated by about 100 km. This suggests that macaws may not necessarily disperse to the nearest colonies over more distant ones. This may also be the consequence of population fragmentation, as several distinct genetic clusters were located within shorter distances of each other (e.g. W2–W4) compared to population nuclei corresponding to the same genetic cluster (e.g. W3). The effect of geographic isolation on genetic isolation appears to be more evident for the southernmost and geographically isolated genetic cluster (W1), which was supported by high assignment values. This was also supported by the lowest number of breeding pairs, a high proportion of non-breeding individuals^30^, and a low effective population size in this population nucleus. Furthermore, while coalescent-based simulations supported a model of drift-gene flow equilibrium for the whole population, the largest identity by descent value, the lowest estimates of contemporary gene flow, and high inbreeding and relatedness indices supported the effects of genetic erosion in the southernmost genetic cluster. These processes can exert a clear influence on a reduction in effective population size and contribute to unequal genetic erosion among population nuclei^37,38^. While a complete divergence among populations should translate into pronounced genetic differences, many private alleles and highly distinct gene clusters, a secondary contact of populations with a rather recent common ancestry should translate to weak differentiation and some level of gene flow^39,40^. This generally occurs because even a small number of individual exchanges may prompt genetic homogenisation^19^. Therefore, the low level of private allelic richness in each genetic cluster, together with some degree of admixture, suggests that the current genetic structure is most likely not the result of genetic fragmentation from a single homogeneous genetic unit in the past. This is supported by the inclusion in the same genetic cluster of breeding colonies separated by relatively large distance. The genetic differentiation observed is only supported by clustering analysis with sample location priors, which may suggest a weak population structure. However, the clustering algorithm with sample location priors assumes that the assignment probabilities to a population of origin vary among locations, not being biased to detect population structure where it does not exist^41^.

Results support that genetic diversity has been probably more affected by historical events than contemporary ones. The lack of a clear significant influence of bottlenecks on heterozygosity-excess tests, except in particular clusters, further indicates a population structure that is not severely affected by genetic diversity loss due to recent demographic processes^42^. On the contrary, the strong evidence of bottlenecks in M-ratio tests clearly support historical (distant past) population contractions eroding genetic diversity across the whole population^43,44^. The M-ratio can detect population size reductions up to approximately 125 generations if the population rebounded quickly after a severe bottleneck event, and up to 500 generations if the population size remains reduced^45^. This suggests that the genetic structure currently observed may likely not be a consequence of genetic erosion in the long-term, because even a reduced gene flow from individuals moving between clusters could have contributed to diluting any structure subsequently, at least during the historical time scale than can be inferred from the study of nuclear markers. Given the approximate generation length estimated for this species (12.7 years according to the IUCN^46^), the historic bottlenecks coincided with drastic habitat fragmentation and transformation in the inter-Andean dry forest of Bolivia by humans during the last Holocene (4000 cal BP—Present), rather than with climatic conditions that were relatively stable during this period^47–49^. Habitat transformation by humans began 3000 years ago and was especially intense during the expansion of the Inca Empire in the fifteenth century, when the inter-Andean valleys were a main source of maize, pasture for llama (*Lama glama*) used for transport, and firewood^47,50,51^. During this period, the persecution of macaws because of their impact on maize crops, and their capture for trade as “prestige goods” and for colourful feathers used in decorative objects and as tributes^52–56^, could have contributed to the historic bottlenecks. Rather than due to human impact, older bottlenecks should be linked to geo-climatic events, which could be inferred by future analysis of mitochondrial DNA.

Overall, the genetic diversity was higher in wild macaws than in captive-bred ones in Spain, but was similar to that observed in individuals maintained in captivity in Bolivia. This was expected, because wild-caught macaws maintained in captivity in Bolivia were captured over the whole range of the species, and founder events should have been limited. Since these individuals do not reproduce in captivity in Bolivia, they cannot be affected by additional inbreeding due to artificial pairing between close relatives, as often occurs in captive-breeding groups^57,58^. In contrast, diversity indices in the captive groups in Spain were particularly low, which suggest that the founder effect due to introduction of only a few individuals 30 years ago in the pet market resulted in a reduced diversity and higher inbreeding levels. In particular, the continued breeding between close relatives in the captive group in Malaga promoted the lowest genetic diversity and its classification in a single genetic cluster (C3), whose origin in wild populations cannot be identified among the sampled nuclei. This population shows the lowest allelic richness value but a high heterozygosity value, which is expected in the first generations after a founder event with a reduced number of individuals and absence of random mating^59,60^. Whether this cluster corresponds to an unsampled population in the wild or emerged as the result of artificial cross-pairing between relatives, it reveals that genetic identity can be maintained even under conditions of high inbreeding. On the contrary, the Bayesian clustering analysis classified captive individuals from a private owner in Cochabamba and those from Loro Parque as corresponding to the wild cluster W4. The captive group in the Zoological Park of Santa Cruz de la Sierra showed a mixed ancestry from two of the genetic clusters in the wild, which indicates that individuals from these clusters were simply maintained together. Our microsatellite panel allowing differentiation and assignment of both wild and captive groups could be a powerful tool for conservation and management actions. Further development of new nuclear markers and the analysis of mitochondrial markers are necessary to improve individual assignment tests and to deepen the knowledge on the diversity and structure of red-fronted macaw populations.

### Drivers of population genetic structure

Genetic structure generally arises as a consequence of species-specific life history traits and their interactions with environmental features like habitat fragmentation and heterogeneity^2,61^, and population characteristics like density and demography^62^. Dispersal patterns have been highlighted to greatly contribute to the distribution of genotypes across geographical areas and distribution ranges^1^. However, even highly mobile terrestrial birds can show low levels of gene flow leading to strong genetic structure^8^. In general, parrots are generalist plant consumers able to make daily, seasonal, and altitudinal long-distance movements by tracking the abundance or preference of food resources^63^. Red-fronted macaws disperse over large areas through long-distance daily flights, including altitudinal movements during the breeding season, and longer seasonal movements to occupy distant foraging areas during the non-breeding season^28–30^. Distances between breeding nuclei forming the genetic clusters observed in the wild population were relatively short for precluding the genetic intermixing of individuals of this vagile species^29,30^. In fact, the analysis of isolation by distance showed a tendency of lower genetic differences at higher geographic proximity, although this was very influenced by the effect of the southernmost isolated genetic cluster. The extent of the altitudinal movements^30^ and the lack of high mountains or unsuitable habitat between genetic clusters indicate that isolation by distance or physical barriers are not important mechanisms limiting gene flow leading to the observed genetic structure. This is further supported by the use of non-breeding areas by flocks larger than those observed in each respectively nearest breeding area or genetic cluster. This suggests the intermixing of individuals from different population nuclei and genetic clusters during temporary associations in large flocks at non-breeding areas, mainly to exploit maize and peanut crops^28–30^. This intermixing should be confirmed by future genetic identification of individuals foraging and communally roosting in each non-breeding area, which would be useful to determine the importance of foraging and roosting sites for each genetic cluster.

Other studies have described a similarly unexpected fine-scale genetic structure not related to dispersal capabilities, but to a combination of ecological, behavioural and/or evolutionary mechanisms^1^. Among drivers of population genetic structure, landscape and ecological features can be major barriers to gene flow between populations^64,65^. This mechanism of isolation by adaptation to specific local conditions does not seem to influence the genetic heterogeneity of the red-fronted macaw population. This is based on the lack of important differences in the location and physical features of the cliffs with breeding colonies and their close surroundings in each genetic cluster. Only altitude of the colonies differed between genetic clusters, although the range of variation is lower than that of the daily and altitudinal movements of macaws^30^. This indicates an effect of the location of cliffs available for colonial nesting in the geographic location of genetic clusters, rather than actual ecological adaptation leading to genetic differentiation. However, among the nuclei not sampled for genetic analysis was a small population of red-fronted macaw nesting on palms^66^, which constitutes an exception that deserves future research for its implications on potential genetic identity associated with nesting habits. In addition, foraging habitat did not differ between population nuclei, and individuals from each particular genetic cluster can extensively move to exploit distant regional and seasonal resources^29,30,67^. In fact, diet composition showed no differences among genetic clusters, as four vegetal species were dominantly exploited across genetic clusters and non-breeding areas throughout the year, while a variety of food species are exploited seasonally at lower frequencies^28,67^. Differences in the frequency of different plants in the diet were even greater between breeding and non-breeding areas than among the breeding areas comprising the genetic clusters, mainly due to the primary exploitation of crops by large flocks in the non-breeding areas. Published models on foraging and habitat selection showed preference for humanized areas (degraded forest patches with crops), but not affected by densities of houses and livestock as proxies of human presence and disturbance^30^. In addition, this species is relatively tolerant in terms of human presence, since it frequently nests and forages in very accessible places and agricultural areas with constant human presence. Therefore, the ecology of macaws in each genetic cluster, as a filter to dispersal and effective interchange between population nuclei, does not support the hypothesis that adaptations to specific local conditions (affected or not by human activities) would reduce performance of dispersers elsewhere.

In the absence of geographical and ecological barriers, isolation by socio-cultural and behavioural factors arises as a reliable mechanism driving fine-scale genetic structure in highly social species^68^. In this context, the spatial distribution of breeding populations is an important social factor in shaping the patterns of genetic diversity throughout a species’ range^69,70^, often associated with and maintained by coloniality, natal or breeding philopatry, and coordinated dispersal of relatives^71^. Several highly vagile and colonial species are faithful to their natal area, which may determine strong genetic structure over small spatial scales despite large-scale movements^8,72,73^. The red-fronted macaw nests in colonies that are repeatedly used across years^28,30,74^, which may favour a patchy distribution of suitable nesting habitat and potential philopatric behaviour of breeding pairs, as is common among parrots^32,75^. Social imprinting to natal colonies may also reinforce philopatry-related genetic structure^32^, but it may act to the detriment of genetic variability by promoting inbreeding^76^. As occurs in other long-lived and highly social species^8,77^, red-fronted macaws from different genetic nuclei can likely flock together in foraging areas and roosting sites used across daily, seasonal and annual long-distance movements^28–30^. The lack of actual genetic intermixing thus arises despite possible intermixing of flocks from different genetic clusters in non-breeding areas used temporarily, which indicates very limited effective dispersal leading to recruitment into non-natal genetic clusters. This can arise by individual recognition within social groups strongly attached to natal and breeding colonies in this species, which may be achieved through complex organisation and behaviour facilitated by informative vocalisations^78^ and dialects common among parrots^16,17,79^.

Evidence of isolation by social behaviour has been suggested in bird species with complex societies, including cooperative breeding and lekking mating systems^7,71^. A combination of strong natal philopatry, close bonds among group members sharing social identity, within-group reproductive skew and kin-cooperation could provide the strong group stability behind pronounced genetic structure observed in these species^7,8,68,71^. A similar combination of factors may concur to explain the genetic structure in the red-fronted macaw irrespective of the potential loss of genetic diversity in particular genetic clusters due to population decline and inbreeding. Few studies have considered this mechanism to explain the unexpected fine-scale genetic structure despite the high mobility of parrots. For instance, social barriers could influence the unexpected fine-scale genetic structure of the cooperative-breeding El Oro parakeet (*Pyrrhura orcesi*)^21^, as complex social organisation in birds can drive decisions about dispersal from family flocks, and the skewed acceptance of dispersers into other groups^7,71^.

Remarkably, the breeding pairs (about 100 in the last census) represent a small proportion (c. 20%) of the whole red-fronted macaw population^30^, as is the case of other colonial cliff-nesting macaw species^80^, which may be indicative of restricted breeding opportunities linked to unfavourable environmental conditions and delayed breeding^81–83^. A non-mutually exclusive hypothesis states that, regardless of environmental limitations, a high proportion of non-breeding individuals can be a consequence of the within-group reproductive skew necessary to maintain the genetic identity of each cluster without losing genetic diversity. In fact, a high proportion of non-breeding individuals is typical of long-lived species with complex societies and pronounced genetic structure, where access to reproduction can depend on familiar and social factors^8,71,82,84^. Therefore, a high proportion of non-breeding individuals in long-lived species with no cooperative breeding could be a species-specific trait evolved to attain enough genetic variability allowing specific pairings among group members of extended family networks, which deserves further research. Inbreeding depression due to artificial pairing between close relatives in captivity represents the opposite process, as it can lead to a specific genetic signature associated with a patent loss of genetic diversity (e.g. cluster C3). More research on genetic diversity and structure of wild and captive populations is required to support this hypothesis, since high inbreeding levels can introduce biased inferences in clustering methods^85,86^.

### Concluding remarks and implications for conservation

Evolutionary history under isolation has been argued to be a major driver of genetic diversity and effective population size especially in rare and threatened species^38^ with geographically-restricted ranges^87^. Factors generally promoting these kinds of genetic and population patterns include geo-climatic events like glaciations isolating small populations in geographically-restricted refugia and habitats^88,89^, as well as the founder effect and extreme population isolation in oceanic islands^90^. The red-fronted macaw adjusts to this evolutionary pattern, as it evolved in isolation in the tropical inter-Andean dry-forest since the rapid uplift that occurred about 10 million years ago in this sector of the Andes range^91^. This event meant the isolation of the relatively small extension of the dry-valleys of Bolivia from the Gran Chaco region, and it was the origin of the high level of plant endemism present in the region^92,93^ with which the red-fronted macaw coevolved^67,94^. The genetic and population characteristics (including the high non-breeder proportion) currently observed in the global population of the red-fronted macaw represent intrinsic features that have evolved as adaptations increasing fitness and population performance under these particular eco-evolutionary conditions. If this hypothesis can be demonstrated as true, this would imply a specific attention on the singular genetic and population structure of this species as key targets for its conservation.

Evidence for a fine-scale structuring in four relatively independent genetic clusters has important implications in the conservation of the whole population of the red-fronted macaw. This species currently shows variable levels of genetic diversity depending on the genetic cluster. However, there is no clear evidence of strong genetic erosion in the population as a whole in the last decades, but rather in the last centuries or millennia. Genetic diversity, reflected in heterozygosity values, is lower than that observed for widely distributed parrots^95^, but similar or higher than those reported for endangered parrot species with restricted distribution ranges or recent population declines^96–98^. However, these results should be interpreted with caution when different markers were used to estimate genetic diversity indices^99^. In addition, low heterozygosity is not always derived from a reduction in effective population size^60^, as demonstrated by the relatively high heterozygosity of the captive cluster C3 despite high actual inbreeding and low allelic richness.

Recent declines due to habitat transformation and human-mediated mortality by persecution of adult birds in agricultural areas and nestling poaching for the local pet trade^30^ have reduced the population size, and could have contributed to the reduction of genetic diversity in some breeding nuclei. The persecution outside breeding areas may further exert an important effect in the reduction of the total and non-breeding population buffering breeder mortality. Of concern is the loss of breeding pairs by direct persecution in the colonies and their surroundings, and by alterations in the nesting habitats, which may exert especially detrimental effects on the effective population size. This is based on the expected concerning effects on population viability and dynamics from adult mortality in long-lived species with deferred maturity and low reproductive rates, as in large macaws^80,100^. In addition, the impact on breeders may greatly reduce genetic diversity under the hypothesis that they harbour most of it as enhanced by highly selective pairings in each genetic cluster. Future investigation should be directed to confirm or reject this hypothesis.

This study emphasises that conservation efforts for the Critically Endangered red-fronted macaw should be focussed on multiple fronts^30^, but especially on the particularly more detrimental threats in each genetic cluster as independent conservation units^101,102^. The restricted range composed of relatively homogeneous habitats may increase extinction risk for the whole population more rapidly through environmental correlation^103^ than due to genetic depression on each particular cluster^26,104^. Therefore, to implement a global conservation strategy preserving these evolutionary units as components of the overall genetic integrity, a comprehensive evaluation is required on the key habitats, home ranges and availability of food resources in each genetic cluster. Special attention should be directed to avoid nestling poaching for the local pet trade and persecution of adults, as key threats with a high impact shared by all population nuclei^30^. Finally, our genetic approach is sufficiently reliable to serve as a reference for future estimations on genetic diversity and potential current or future demographic bottlenecks in each genetic cluster. Future genetic analysis of the three wild breeding nuclei not sampled in this study will serve to determine the potential existence of additional clusters with a singular genetic identity. This will also help to better identify the origin of captive individuals, and those admitted in recovery centres after seizure from illegal trade. The assignment of captive individuals to each genetic cluster may be useful to form suitable captive-breeding nuclei for future potential reinforcement programs and reintroduction of individuals in their corresponding genetic nuclei in the wild.

## Materials and methods

### Study area and study species

The red-fronted macaw is a colonial, cliff-nesting species endemic to the inter-Andean valleys of Bolivia. This is a wide, rugged and dry area located on the eastern slopes of the central Bolivian Andes at altitudes from 900 to 3500 m, in the departments of Santa Cruz, Cochabamba, Chuquisaca, and Potosi^30^. The habitat is dominated by tropical dry forest with a high proportion of endemism, transformed by long-term human activities to thorn and cactus scrub with scattered trees in hillsides and riverine forest and crops in valley bottoms^92^.

The small population size, reduced distribution range, and recent decline led to the listing of the species as “Critically Endangered” according to the IUCN Red List^46^. Population estimates varied from 5000 individuals in the 1980s to 2000–4000 or as few as 700–800 individuals and less than 100 breeding pairs in recent years^30,46^. Forest loss and degradation due to agriculture expansion, overgrazing and firewood cutting, and poaching of nestlings for local pets, are considered the major threats to the species^30,46^.

### Fieldwork

This genetic approach benefits from previous fieldwork conducted for assessing the global distribution and population size of the species, its seasonal movements, diet and foraging habits as detailed in our previous publications^30,67,94^. Briefly, all known breeding colonies and another found for first time, as well as communal roosting sites, were simultaneously monitored in 2011, allowing us to estimate the global population size (c. 800 individuals), with c. 100 pairs breeding in 8 discrete areas (summing 1298 km^2^, 114–300 km^2^ each, see Fig. 1a), and a non-breeding population reaching c. 80% of the total^30^. To characterise the breeding colonies, we recorded the nesting substrate, height, width (in m), orientation (transformed to radians for statistical analysis), altitude (in m above sea level), and distance (in m) to the nearest river of the cliffs holding breeding colonies^30^. Breeding nuclei are located in river cliffs. The population size was much larger in the recent past^30^, and thus in the last decades some breeding cliffs have been abandoned. For instance, in the first more complete census of the species, it had already disappeared as breeder of six cliff areas, while it was found breeding in 12 new cliffs where its reproductive presence was not known before^30^. This species nests in a wide altitude range, where we found another 81 cliffs not occupied by the species, while in 41 of them nested up four other parrot species that usually nest coexisting with the red-fronted macaw in the same cliffs. Therefore, we can assume that at least a proportion of these cliffs could be suitable for macaws according to their features, similar to those of used cliffs. Therefore, the availability of cliffs is apparently not a limiting factor, although there is no more data on breeding areas than known after intensive searching by us, other research teams, and personnel of local governments, especially in protected areas.

Seasonal movements were studied in 2011–2012 (588 flocks recorded), showing changes in flock size and movements of macaws between 953 and 3094 m a.s.l. that increased after the breeding season to occupy six non-breeding foraging areas (summing 1329 km^2^, each range 61–459 km^2^, see Fig. 1a)^30^. The foraging ecology and diet of the species was studied year-round during 2011–2013, through roadside surveys (totalling 6823 km) conducted during 153 fieldwork days in eight expeditions, which allowed us to record 220 foraging flocks (summing c. 2500 individuals) and the plant species they were consuming^67,94^.

Samples of blood and feathers were collected from both wild and captive individuals in Bolivia and from captive individuals in Spain (Table 3). The sampling area covered the whole distribution of the species in the wild, although colonies from a few areas were not sampled due to very difficult access (Fig. 1). Field surveys were optimised in space to ensure the sampling of individuals from previously undetected populations^30^. A total of 114 wing and tail feathers were collected from five areas (Table 3) during field surveys conducted in 2012. All feathers were sampled at the end of the breeding period beneath the cliffs with breeding colonies and their surroundings, and in communal roosts close to the colonies (Table 3). Blood samples were obtained from five nestlings poached in a breeding area (code D in Table 3, Fig. 1) and five adults recently captured in a non-breeding area (code G in Table 3, Fig. 1).

Regarding the captive populations, blood samples were obtained in 2012 from the Zoo of Santa Cruz de la Sierra (ZOOL, *n* = 12) and from a private owner in Cochamamba (COCH, *n* = 12) in Bolivia. These macaws were captured within the last ten years from the wild population (pers. comm. from the keepers). Feathers (*n* = 36) and blood (*n* = 37) samples from captive individuals in Spain were obtained from different private owners (Table 3). All captive macaws in Malaga originated from six individuals from the pet trade 30 years ago in England (Lesley Munns, pers. comm.), while the rest were purchased at two public zoos (Loro Parque and Palmitos Park).

### Microsatellite genotyping and individual identification

Genomic DNA was extracted from blood and feather samples. All individuals were genotyped using a microsatellite panel of 9 polymorphic loci (UnaCT21, UnaCT32, UnaCT43, UnaCT74, AgGT17, AgGT21, AgGT90, MmGT057 and Peeμ11) specifically selected for this study (see Supplementary Methods S1 for details; Table S2). Error rates were estimate from replicates of nine low quality DNA samples extracted from feathers. Samples were amplified two or three times (the third amplification was only performed when validation of allele sizes was needed) and only alleles with at least two successfully amplifications were included in analysis. Individuals missing four or more loci were excluded from the final data set. Probability of identity values (*P*_ID_) for each locus were estimated using the program GIMLET 1.3.3^105^.

To detect samples of the same individual and potential family relatives, individuals from each sampling site were screened for close genetic relatedness (i.e. total match of all alleles or presence of full-sibs) using COLONY version 2.0.6.6^106^. The analysis was performed considering error rates of the data set, applying the full-likelihood method, long length of run, medium-likelihood precision, and no sibship prior, assuming a monogamous mating system and an inbreeding model for isolated populations^107^. Full-sib pairs with a probability > 0.90 detected after four long runs were considered related familiar sampling. This sibship reconstruction is essential to assess the quality of our data set, but we cannot exclude some potential bias in the inferences, since we do not know the birth year of individuals and we do not have family groups from any nucleus to calibrate the inference methods. Consequently, we cannot exclude that some individuals identified as full sibs may be parent-offspring. Molecular sex identification was considered as an additional criterion to assign a given consensus genotype to the same or different individuals (Supplementary Methods S1).

### Bayesian clustering analysis and population genetic differentiation

We assessed pairwise genetic differentiation between red-fronted macaw populations with the *F*_*st*_ estimator of Weir and Cockerham^108^, as implemented in GENALEX v.6.5^109^. To further explore genetic evidence for subdivision among individuals (both wild and captive), the partition of the total genetic variation was explored based on a Principal Coordinate Analysis (PCoA) in GENALEX. Further, to be able to identify the actual subpopulations and assign individuals (probabilistically) to these populations, we used Bayesian analysis implemented in the program STRUCTURE v.2.1^110^. We used the model of correlated allele frequencies and admixture^110,111^. We ran 20 replicates for each *K* in the range of 1 to 8 in order to estimate the most likely number of clusters (*K*), using all individuals. Simulations were conducted with 1 000 000 steps of the Markov chain Monte Carlo (MCMC) after a burn-in period of 100 000 iterations without and with sampling localities as priors (LOCPRIOR option) to allow detection of weaker genetic population structure^41^. The outputs were processed in the CLUMPAK server^112^ to display the results in a graphical interface and to obtain assignment probabilities of individuals (*q*) to each cluster (using a MCL threshold for similarity scores of 0.90). The likelihood distributions were analysed with STRUCTURE HARVESTER^113^. The optimal *K* value was selected considering the following assumptions: (1) the likelihood distribution reached a maximum and began to plateau or decrease; (2) high stability of clustering patterns between runs (at least 60% of the 20 runs were similar in the primary mode); and (3) *K*_max_ + 1 no longer identify new clusters (i.e. the genetic structure at *K*_max_ + 1 is equal to *K*_max_)^8,114^. We also estimate the *K* value for each analysis using the method reported by Evanno et al. (2005)^115^. We tested a hierarchical STRUCTURE approach to search for population structure not detected at the first level of the STRUCTURE analysis^8,115^.

### Genetic diversity

Diversity parameters were calculated for wild and captive populations in Bolivia, as well as for the two captive groups from Spain (Table 3). Departures from linkage disequilibrium and Hardy–Weinberg equilibrium (HWE) were tested using exact tests as implemented in GENEPOP v.4.4.2^116^, applying the Markov chain method with 10,000 dememorisation steps, 1000 batches and 5000 iterations/batch. Bonferroni corrections to significance values were applied to account for multiple tests. Genetic diversity (i.e. observed (*H*_*O*_) and expected heterozygosity (*H*_*E*_), and mean number of alleles per locus (*N*_*A*_)) was estimated for each locus and population using GENALEX v.6.5. The program FSTAT v.2.9.4 was used to calculate allelic richness (*A*_*R*_). Polymorphism information content (PIC), a measure of the informativeness degree of a marker used as a key parameter to evaluate the ability to detect genetic variation among individuals of a population^117^, was calculated for all loci per population using CERVUS v.3.0.7^118^.

### Drift versus gene flow model

We determined the relative contributions of drift versus gene flow across the distribution area, using the likelihood approach implemented in the program 2MOD^119^. Two models were compared: the drift model relates to populations that are subject to drift alone, with no influence from gene flow, while the gene flow/drift equilibrium model (referred to as the gene flow model) relates to a balance between the two forces. The drift model assumes that mutation has not strongly influenced gene frequencies such that alleles are identical by descent, and the gene flow model assumes that the mutation rate is much smaller than the immigration rate^119^. We carried out five independent runs to ensure convergence of the MCMC algorithm, with 1000 000 iterations and the first 10% discarded as burn-in. The probability of a model was calculated as the proportion of times that the model was supported. The number of migrants per generation (M) for each population was calculated from F values according to Ciofi et al.^119^.

### Demographic inferences, inbreeding, relatedness and drivers of genetic differentiation

To test for possible reductions in population size, we used the program BOTTLENECK v.1.2.02^120^ and the M-ratio model implemented in the software M_P_VAL^45^ (details in Supplementary Methods S1).

FSTAT was used to explore the level of inbreeding in each population using the inbreeding coefficient (*F*_*IS*_). Mean relatedness within each breeding site was estimated in the program GENALEX, using Queller and Goodnight’s R estimates^121^.

To estimate recent migration rates between groups (i.e., within the last *N*_*e*_ generations, where *N*_*e*_ is the effective population size) in each direction and rates of dispersal, we used a Bayesian approach (BAYESASS v.1.2^122^), and the program GENECLASS2 v.2.0^123^, a different approach to detect first generation migrants. See the Supplementary Methods S1 for a detailed explanation of these program settings.

To estimate the contemporary effective population size (*N*_*e*_) of the whole population, as well as for each breeding colony, we applied the linkage disequilibrium method using the program LDNE v.1.31^124^. The LD method provides values of the *N*_*e*_ in the parental generation of the individuals analysed, but the precision of the estimates can be affected if occurred changes in population sizes in the recent past^124^. This program assumes random mating and excludes all alleles with frequencies lower than 0.02, which generally provides a good balance between precision and bias with highly polymorphic microsatellite loci^125^. The jack-knife procedure was selected to obtain the confidence intervals (CIs).

Patterns of isolation-by-distance (IBD) arise when population differentiation increases with increasing geographic distances, and are usually caused by local spatial dispersal. We analysed IBD by regressing pairwise estimates of *F*_*st*_ against distance in kms between breeding sites^126^. We used Mantel tests to test the correlation between matrices of genetic differentiation and geographic distances by 1000 permutations in IBD program v.1.52^127^.

### Ethics statements

All fieldwork and procedures, including moulted feather collection and blood sampling conducted in Bolivia were carried out in accordance with relevant guidelines and regulations to study endangered species by Viceministerio de Medio Ambiente, Biodiversidad y Cambios Climáticos of Bolivia (18052012) in collaboration between Museo de Historia Natural Noel Kempff Mercado (Bolivia) and Estación Biológica de Doñana, Consejo Superior de Investigaciones Científicas (CSIC, Spain), and in accordance with the approved guidelines of the Consejo Superior de Investigaciones Científicas (CSIC, Spain). Sampling protocols used in this study were approved by the Ethic Committee of the Consejo Superior de Investigaciones Científicas, CSIC (CEBA-EBD_11_27). The samples obtained in Bolivia were exported to Spain for molecular analyses under CITES permit no. 01998.

## Supplementary Information

Supplementary Information 1.

Supplementary Information 2.

Supplementary Information 3.

## Data Availability

All data generated or analysed during this study are included in this published article (and its Supplementary Information files).
