# Supplementary material for: Fine-scale genetic structure in the critically endangered red-fronted macaw in the absence of geographic and ecological barriers
Source: Sci Rep. 2021 Jan 12;11:556. doi: 10.1038/s41598-020-79575-6 (PMC7804180; doi:10.1038/s41598-020-79575-6)
Supplement: Supplementary file 2 — Supplementary Information 2. [file 41598_2020_79575_MOESM2_ESM.docx]

Supplementary Information

**Fine-scale genetic structure in the critically endangered red-fronted macaw in the absence of geographic and ecological barriers**

Guillermo Blanco, Francisco Morinha, Séverine Roques, Fernando Hiraldo, Abraham Rojas, José L. Tella^3^

**Supplementary Methods**

**DNA extraction**

Blood samples were preserved in absolute ethanol and kept at 4°C until their processing in the laboratory. DNA isolation from blood followed standard phenol–chloroform extraction protocols^1^. Feathers were kept at -20^o^C until extraction. DNA was extracted following the method of Bello et al.^2^ in a laboratory free from PCR (polymerase chain reaction) products and specially designated for the manipulation of non-invasive material.

**Microsatellite genotyping**

We tested 22 microsatellite loci previously developed for other parrot species (UnaCT21, UnaCT32, UnaCT43, UnaGT55, UnaCT74; AgGT17, AgGT19, AgGT21, AgGT42, AgGT90; AgGT02, AgGT04, AgGT07, AgGT08, AgGT12, AgGT21, AgGT81, AgGT83; MmGT030, MmGT057; Peeµ11, Peeµ16)^3,4,5,6^ using single PCR amplifications of DNA from blood samples. The selection of the microsatellite panel for red-fronted macaw took into account the efficiency of PCR amplification, genotype reliability (allelic dropout and false alleles, computed using GIMLET v.1.3.3^7^), multiplexing compatibilities and genetic variability indices (allelic diversity, observed and expected heterozygosity) calculated using GENETIX v.4.05^8^ and GIMLET v.1.3.3. After a careful evaluation of all parameters and statistics, a set of 9 markers were selected (UnaCT21, UnaCT32, UnaCT43, UnaCT74, AgGT17, AgGT21, AgGT90, MmGT057 and Peeμ11) (Table S2). For blood samples, we performed a single multiplex (i.e. MPLX) reaction of these 9 loci using the “Type-it microsatellite kit” from QIAGEN. For low quality samples (i.e. feathers), amplification conditions were optimised on two extraction replicates of three feathers from a known captive individual, for which we also obtained a blood sample, as a positive control. We followed a serial strategy depending on the DNA concentrations estimated using a Nanodrop instrument (Thermo Scientific). We first amplified all extracts with sufficient quantity of DNA (≥ 5 ng/ul) using the MPLX protocol. For those with lower quantity (< 5 ng/ul), we used the MPLX PCR as a template for a subsequent PCR in which a single locus was amplified per reaction. DNA of those feathers was amplified between 2 and 3 times to ensure genotyping consistency. PCR amplification was carried out in 25 μl reaction volumes using an ABI 480 thermocycler (Perkin-Elmer; Foster City, CA, USA) with approximately 50 ng of genomic DNA as template. Final amplification conditions consisted of 12.5 pmol unlabelled reverse primer, 12.5 pmol fluorescently labelled forward primer, 1.5 mM MgCl2, 200 μM each dNTP, and 0.5 units of Taq DNA polymerase (Promega; Madison, WI, USA). The thermal profile for PCR amplification was 95°C for 5 min, followed by 35 cycles of denaturing at 95°C for 30 s, annealing at a primer-specific temperature for 30 s, and elongated at 72°C for 30 s, ending with a single extension of 72°C for 10 min.

**Molecular sexing**

Molecular sexing was performed based on the chromodomain helicase DNA-binding

protein 1 (*CHD1*) gene, using the primers M5 (5'-YTYMCTTCAYTTCCATTAAAGC-3') and P8 (5'-CTCCCAAGGATGAGRAAYTG-3')^9,10^. PCR consisted of 4 μl of DNA extract in a final volume of 25 μl, containing 10-50 ng DNA, 1 U Taq polymerase (Promega; Madison, WI, USA), 1 x PCR buffer (NH4)2SO4, 1.5 mM MgCl2, 200 μM each dNTP, and 25 pM of each primer. Cycling steps included a first denaturation step at 94ºC for 5 minutes, followed by 40 cycles of denaturation at 94ºC, annealing at 58ºC and extension at 72ºC, each step lasting 30 seconds, and a final extension step of 5 min at 72ºC.The PCR products were run on 2.5 % agarose gels. Individuals with a total match of all genotypes and the same sex were excluded from the final data set used in the study.

**Bottleneck analysis**

BOTTLENECK detects whether there is an excess or deficiency of heterozygotes relative to expected heterozygosity under mutation–drift equilibrium. The expected heterozygosity under Hardy–Weinberg equilibrium is higher than the expected heterozygosity under mutation-drift equilibrium in recently bottlenecked populations^11,12,13^. The stepwise mutation model (SMM), infinite allele model (IAM) and two-phase mutation (TPM) model (30%, variance for TPM and 95%, proportion of SMM in TPM) was tested with 10 000 iterations. Significance was determined with a Wilcoxon signed-rank test, which gives the highest statistical power when population sample size is small (30 or fewer)^11^. The mode-shift test was also applied^12^. Following a bottleneck, it is expected that the allele frequency distribution will have shifted towards more common alleles with fewer low-frequency alleles. The M-ratio model assumes that during population bottlenecks, the total number of alleles in a population (k) tends to decrease faster than the total number of expected alleles considering the allelic size range (r). The ratio (k/r) tends to be smaller in recently bottlenecked populations when compared to populations in equilibrium (Mc). The critical values (Mc) were calculated with the program CRITICAL_M^14^. The analyses were carried out using the average repetition frequency of multi-step mutation (Δg) = 3.1 and proportions of multi-step mutations (Pg) = 0.22 (as recommended by Peery et al.^15^ to minimise the probability of type I errors), testing three different values of θ (0.1, 1 and 10). The empirical value of the M-ratio was compared to the bottleneck threshold (0.68) suggested by Garza and Williamson^14^, as well as the Mc values obtained for all population clusters to assess the significance of past bottleneck events.

**Estimation of migration rates**

BAYESASS simultaneously estimates recent migration rates along with individual migrant ancestries: first migrant (i.e. individuals that have moved from one population to another) and second generation migrants (i.e. offspring of a migrant and a non-migrant)^16^. Whether the occurrence of first migrants indicates dispersal, second generation migrants would be indicative of gene flow. Further, BAYESASS permits the assessment of the proportions of individuals assigned to their hypothesised population and to other populations. The analysis consisted of five independent runs of 2 × 108 iterations (with a burn-in of 2 × 107 iterations) and a sampling frequency of 1000. The mixing parameters were adjusted (ΔM = 0.1; ΔF = 0.5; ΔA = 0.3) and replicate runs were performed with different starting seed values to check the consistency of the results.

In GENECLASS2, each individual is assigned to the most probable population of its origin based on its microsatellite genotype. Population assignments were carried out similarly to BAYESASS, using K = 6 for initial population assignments. Both tests were performed using the Rannala method and Monte-Carlo resampling algorithm of Paetkau et al.^17^ with 10 000 simulated individuals and a threshold of 0.01 to calculate the assignment probabilities. The comparison of the number of first generation migrants between populations (i.e. obtained from BAYESASS and GENECLASS) and second generation migrants (i.e., obtained from BAYESASS) allowed us to distinguish dispersal from gene flow.

**Supplementary Tables**

**Table S1.** Probabilities of identity obtained for the nine microsatellite loci used to genotype red-fronted macaws.

| **Locus** | ***P*_(ID)_** | ***P*_(ID)sib_** |
| --- | --- | --- |
| UnaCT21 | 0.2452 | 0.5509 |
| UnaCT32 | 0.1160 | 0.4225 |
| UnaCT43 | 0.0415 | 0.3420 |
| UnaCT74 | 0.6752 | 0.8301 |
| AgGT17 | 0.3240 | 0.5856 |
| AgGT21 | 0.0449 | 0.3515 |
| AgGT90 | 0.0665 | 0.3708 |
| MmGT057 | 0.4739 | 0.6960 |
| Peeμ11 | 0.0734 | 0.3804 |

**Table S2.** Previously published microsatellite loci successfully applied in the red-fronted macaws.

| **Locus** | **Originally developed for** | **Repeat sequence** | **Primer sequences (5′–3′)** | **Reference** |
| --- | --- | --- | --- | --- |
| UnaCT21 | *Ara ararauna; Ara chloropterus*; *Ara macao*; *Ara severus*; *Orthopsittaca manilata; Amazona farinosa*. | (GT)_n_(CTT)(GT)_n_ | F: AAACGAGATGACCACCGAAA  R: TAACTCCCTCCAGCCAGCTT | Gebhardt and Waits, 2008 |
| UnaCT32 | *Ara ararauna; Ara chloropterus*; *Ara macao*; *Ara severus*; *Orthopsittaca manilata; Amazona farinosa*. | (GT)_n_ | F: CAGGAAGCACGGAAACAAAT  R: TGCTTTTGTGTCTGCTTTGG | Gebhardt and Waits, 2008 |
| UnaCT43 | *Ara ararauna; Ara chloropterus*; *Ara macao*; *Ara severus*; *Orthopsittaca manilata; Amazona farinosa*. | (GT)_n_ | F: AGTGCAGAGGGGAATTCAGA  R: AAGTCAGTCAACGCTTCAACA | Gebhardt and Waits, 2008 |
| UnaCT74 | *Ara ararauna; Ara chloropterus*; *Ara macao*; *Ara severus*; *Orthopsittaca manilata; Amazona farinosa*. | (GT)_n_ | F: TGCTGCTCTTAACATTTCCTGA  R: GCAGAGTTGCATTTCATTTCTC | Gebhardt and Waits, 2008 |
| AgGT17 | *Ara ararauna; Ara chloropterus*; *Ara macao*; *Ara severus*; *Orthopsittaca manilata; Amazona farinosa*. | (GT)_n_ | F: AACTGCATGGGTTCAAGCTC  R: AGCTGGTTTTGTTCCGAAAG | Gebhardt and Waits, 2008 |
| AgGT21 | *Amazona guildingii* | (GT)n | F: TCCCAGGCCAACACATTTAC  R: GCTTAGTGCATATCCCAAGCTA | Russello et al. 2001 |
| AgGT90 | *Ara ararauna; Ara chloropterus; Ara macao; Ara severus; Orthopsittaca manilata; Amazona farinosa.* | (GT)_n_ | F: TCCACAATTCTACCGAAGTGG  R: ACAGACACATCACTCCCCAAC | Gebhardt and Waits, 2008 |
| MmGT057 | *Myiopsitta monachus* | (CA)_n_(AA)(CA)_n_ | F: TCACTGAGCCTTGAACACATCT  R: ACCAGACCCGCCTTTTGTAT | Russello et al. 2007 |
| Peeμ11 | *Psittacus erithacus* | (CCAT)_n_ | F: AGATGCAAGGAATGTTAAACAC  R: CTCTGCTGCTAGGATAGTTC | Taylor and Parkin 2007 |

**Table S3.** Summary of population genetics data.

A) Locus (UnaCT21, UnaCT32 and UnaCT43)

|  | **UnaCT21** | | | | | | |  | **UnaCT32** | | | | | | |  | **UnaCT43** | | | | | | |
| --- | --- | --- | --- | --- | --- | --- | --- | --- | --- | --- | --- | --- | --- | --- | --- | --- | --- | --- | --- | --- | --- | --- | --- |
| **Pop** | ***N_A_*** | ***A_R_*** | ***H_O_*** | ***H_E_*** | ***P*** | ***F_IS_*** | **PIC** |  | ***N_A_*** | ***A_R_*** | ***H_O_*** | ***H_E_*** | ***P*** | ***F_IS_*** | **PIC** |  | ***N_A_*** | ***A_R_*** | ***H_O_*** | ***H_E_*** | ***P*** | ***F_IS_*** | **PIC** |
| W1 | 4 | 3.35 | 0.25 | 0.28 | 0.31 | 0.13 | 0.26 |  | 5 | 4.73 | 0.67 | 0.71 | 0.78 | 0.10 | 0.67 |  | 4 | 3.97 | 0.69 | 0.63 | 0.95 | -0.06 | 0.58 |
| W2 | 4 | 3.74 | 0.36 | 0.36 | 0.49 | 0.06 | 0.34 |  | 5 | 4.57 | 0.79 | 0.68 | 1.00 | -0.12 | 0.62 |  | 7 | 6.14 | 0.86 | 0.74 | 0.50 | -0.12 | 0.70 |
| W3 | 5 | 4.68 | 0.63 | 0.63 | 0.73 | 0.02 | 0.60 |  | 7 | 4.85 | 0.80 | 0.70 | 0.84 | -0.12 | 0.65 |  | 7 | 6.07 | 0.80 | 0.82 | 0.40 | 0.03 | 0.79 |
| W4 | 4 | 3.16 | 0.37 | 0.48 | 0.47 | 0.26 | 0.41 |  | 6 | 5.17 | 0.68 | 0.68 | 0.69 | 0.02 | 0.62 |  | 8 | 7.26 | 0.83 | 0.82 | 0.51 | 0.02 | 0.80 |
| C1 | 4 | 3.98 | 0.37 | 0.60 | 0.86 | -0.11 | 0.55 |  | 5 | 4.83 | 0.68 | 0.61 | 0.77 | -0.09 | 0.57 |  | 5 | 4.85 | 0.83 | 0.75 | 0.58 | -0.20 | 0.71 |
| C2 | 4 | 3.93 | 0.67 | 0.59 | 0.11 | -0.10 | 0.54 |  | 5 | 4.56 | 0.72 | 0.66 | 0.28 | -0.07 | 0.61 |  | 7 | 6.70 | 0.72 | 0.81 | 0.82 | 0.14 | 0.78 |
| C3 | 4 | 4.00 | 0.90 | 0.74 | 0.10 | -0.20 | 0.69 |  | 2 | 2.00 | 0.58 | 0.49 | 0.65 | -0.15 | 0.37 |  | 4 | 3.52 | 0.90 | 0.64 | 0.12 | -0.39 | 0.57 |
|  |  |  |  |  |  |  |  |  |  |  |  |  |  |  |  |  |  |  |  |  |  |  |  |
| **Mean** | 4.14 | 3.84 | 0.51 | 0.53 | - | 0.01 | 0.49 |  | 5.00 | 4.39 | 0.70 | 0.65 | - | -0.06 | 0.59 |  | 6.00 | 5.50 | 0.81 | 0.74 | - | -0.08 | 0.70 |
|  |  |  |  |  |  |  |  |  |  |  |  |  |  |  |  |  |  |  |  |  |  |  |  |

B) Locus (UnaCT74, AgGT17 and AgGT21)

|  | **UnaCT74** | | | | | | |  | **AgGT17** | | | | | | |  | **AgGT21** | | | | | | |
| --- | --- | --- | --- | --- | --- | --- | --- | --- | --- | --- | --- | --- | --- | --- | --- | --- | --- | --- | --- | --- | --- | --- | --- |
| **Pop** | ***N_A_*** | ***A_R_*** | ***H_O_*** | ***H_E_*** | ***P*** | ***F_IS_*** | **PIC** |  | ***N_A_*** | ***A_R_*** | ***H_O_*** | ***H_E_*** | ***P*** | ***F_IS_*** | **PIC** |  | ***N_A_*** | ***A_R_*** | ***H_O_*** | ***H_E_*** | ***P*** | ***F_IS_*** | **PIC** |
| W1 | 2 | 1.69 | 0.06 | 0.06 | 1.00 | 0.00 | 0.06 |  | 3 | 2.68 | 0.31 | 0.27 | 1.00 | -0.12 | 0.25 |  | 7 | 6.84 | 0.80 | 0.82 | 0.02 | 0.06 | 0.79 |
| W2 | 2 | 2.00 | 0.36 | 0.29 | 1.00 | -0.18 | 0.25 |  | 2 | 2.00 | 0.36 | 0.50 | 0.32 | 0.32 | 0.37 |  | 7 | 6.35 | 0.64 | 0.69 | 0.56 | 0.11 | 0.66 |
| W3 | 2 | 1.76 | 0.11 | 0.10 | 0.56 | -0.05 | 0.10 |  | 4 | 3.11 | 0.49 | 0.48 | 0.78 | 0.00 | 0.42 |  | 11 | 7.19 | 0.81 | 0.78 | 0.13 | -0.03 | 0.75 |
| W4 | 3 | 2.91 | 0.31 | 0.36 | 1.00 | 0.17 | 0.33 |  | 3 | 2.98 | 0.67 | 0.59 | 0.07 | -0.11 | 0.50 |  | 8 | 7.41 | 0.78 | 0.84 | 0.18 | 0.10 | 0.82 |
| C1 | 2 | 2.00 | 0.31 | 0.31 | 1.00 | -0.20 | 0.26 |  | 2 | 2.00 | 0.67 | 0.26 | 1.00 | -0.14 | 0.23 |  | 6 | 5.39 | 0.78 | 0.53 | 0.41 | 0.02 | 0.49 |
| C2 | 2 | 1.95 | 0.17 | 0.15 | 0.53 | -0.06 | 0.14 |  | 3 | 3.00 | 0.50 | 0.60 | 0.15 | 0.20 | 0.53 |  | 12 | 9.78 | 0.94 | 0.84 | 0.64 | -0.10 | 0.82 |
| C3 | 2 | 2.00 | 0.43 | 0.34 | 1.00 | -0.25 | 0.28 |  | 2 | 2.00 | 0.71 | 0.48 | 0.07 | -0.46 | 0.37 |  | 3 | 2.95 | 0.48 | 0.39 | 1.00 | -0.20 | 0.36 |
|  |  |  |  |  |  |  |  |  |  |  |  |  |  |  |  |  |  |  |  |  |  |  |  |
| **Mean** | 2.14 | 2.04 | 0.25 | 0.23 | - | -0.08 | 0.20 |  | 2.71 | 2.54 | 0.53 | 0.45 | - | -0.05 | 0.38 |  | 7.71 | 6.56 | 0.75 | 0.70 | - | -0.01 | 0.67 |
|  |  |  |  |  |  |  |  |  |  |  |  |  |  |  |  |  |  |  |  |  |  |  |  |

C) Locus (AgGT90, MmGT057 and Peeμ11)

|  | **AgGT90** | | | | | | |  | **MmGT057** | | | | | | |  | **Peeμ11** | | | | | | |
| --- | --- | --- | --- | --- | --- | --- | --- | --- | --- | --- | --- | --- | --- | --- | --- | --- | --- | --- | --- | --- | --- | --- | --- |
| **Pop** | ***N_A_*** | ***A_R_*** | ***H_O_*** | ***H_E_*** | ***P*** | ***F_IS_*** | **PIC** |  | ***N_A_*** | ***A_R_*** | ***H_O_*** | ***H_E_*** | ***P*** | ***F_IS_*** | **PIC** |  | ***N_A_*** | ***A_R_*** | ***H_O_*** | ***H_E_*** | ***P*** | ***F_IS_*** | **PIC** |
| W1 | 8 | 7.30 | 0.69 | 0.76 | 0.03 | 0.13 | 0.74 |  | 2 | 1.98 | 0.19 | 0.17 | 1.00 | -0.07 | 0.16 |  | 5 | 5.00 | 0.46 | 0.79 | 0.04 | 0.45 | 0.76 |
| W2 | 6 | 6.00 | 0.73 | 0.78 | 0.55 | 0.11 | 0.74 |  | 2 | 2.00 | 0.43 | 0.46 | 1.00 | 0.10 | 0.35 |  | 5 | 4.85 | 0.54 | 0.70 | 0.11 | 0.26 | 0.65 |
| W3 | 6 | 5.01 | 0.62 | 0.73 | 0.07 | 0.17 | 0.69 |  | 3 | 2.25 | 0.26 | 0.32 | 0.30 | 0.22 | 0.28 |  | 7 | 5.34 | 0.79 | 0.71 | 0.70 | -0.11 | 0.67 |
| W4 | 6 | 5.90 | 0.50 | 0.81 | <0.01 | 0.41 | 0.78 |  | 2 | 2.00 | 0.58 | 0.41 | 0.25 | -0.39 | 0.33 |  | 5 | 4.61 | 0.76 | 0.71 | 0.35 | -0.05 | 0.66 |
| C1 | 6 | 5.69 | 0.50 | 0.73 | 0.12 | 0.20 | 0.69 |  | 3 | 2.85 | 0.58 | 0.32 | 1.00 | -0.15 | 0.29 |  | 5 | 4.85 | 0.76 | 0.73 | 0.41 | -0.23 | 0.68 |
| C2 | 5 | 4.88 | 0.94 | 0.75 | 0.07 | -0.22 | 0.71 |  | 2 | 2.00 | 0.28 | 0.31 | 0.51 | 0.14 | 0.26 |  | 6 | 5.22 | 0.94 | 0.75 | 0.02 | -0.23 | 0.71 |
| C3 | 6 | 4.75 | 0.81 | 0.60 | 0.02 | -0.34 | 0.53 |  | 2 | 1.55 | 0.05 | 0.05 | 1.00 | 0.00 | 0.05 |  | 4 | 3.52 | 0.81 | 0.68 | 0.24 | -0.17 | 0.61 |
|  |  |  |  |  |  |  |  |  |  |  |  |  |  |  |  |  |  |  |  |  |  |  |  |
| **Mean** | 6.14 | 5.65 | 0.68 | 0.74 | - | 0.07 | 0.70 |  | 2.29 | 2.09 | 0.34 | 0.29 | - | -0.02 | 0.25 |  | 5.29 | 4.77 | 0.72 | 0.72 | - | -0.01 | 0.68 |
|  |  |  |  |  |  |  |  |  |  |  |  |  |  |  |  |  |  |  |  |  |  |  |  |
| *N_A_* - number of alleles, *H_O_* - observed heterozygosity, *H_E_* - expected heterozygosity; *A_R_* - Allelic richness; *P* - exact probability for expected Hardy Weinberg equilibrium conditions for each locus/population combination,  *F_IS_* – inbreeding coefficients; PIC - polymorphism information content. **Values in bold:** deviations from HWE significant after Bonferroni correction. | | | | | | | | | | | | | | | | | | | | | | | |

**Table S4.** Frequency of private alleles by genetic cluster in the red-fronted macaw.

| Genetic cluster | Locus | Allele | Frequency |
| --- | --- | --- | --- |
| W1 | Peu11 | 334 | 0.192 |
| W2 | CT43N | 169 | 0.036 |
| W2 | CT32 | 159 | 0.036 |
| C2 | GT21 | 277 | 0.083 |
| C2 | GT21 | 279 | 0.083 |
| C3 | GT90 | 196 | 0.031 |

**Table S5.** Characteristics of the breeding colonies in each genetic cluster of red-fronted macaw in the wild.

|  |  |  |  |  |  |  | Kruskal-Wallis test | | |
| --- | --- | --- | --- | --- | --- | --- | --- | --- | --- |
| Variable | Genetic cluster | *n* | Mean | SD | Minimum | Maximum | H | df | p |
| Altitude | W1 | 5 | 2177.0 | 101.3 | 2050 | 2300 |  |  |  |
|  | W2 | 8 | 1525.0 | 36.2 | 1500 | 1600 |  |  |  |
|  | W3 | 20 | 1843.0 | 251.7 | 1640 | 2640 |  |  |  |
|  | W4 | 9 | 1404.9 | 189.0 | 1272 | 1801 |  |  |  |
|  | Total | 42 | 1728.3 | 315.5 | 1272 | 2640 | 29.38 | 3 | <0.0001 |
| Height | W1 | 5 | 48.0 | 8.4 | 40 | 60 |  |  |  |
|  | W2 | 8 | 45.0 | 21.4 | 30 | 80 |  |  |  |
|  | W3 | 20 | 75.3 | 38.8 | 25 | 200 |  |  |  |
|  | W4 | 9 | 56.1 | 14.5 | 25 | 70 |  |  |  |
|  | Total | 42 | 62.1 | 31.6 | 25 | 200 | 7.553 | 3 | 0.056 |
| Width | W1 | 5 | 174.0 | 79.9 | 100 | 300 |  |  |  |
|  | W2 | 8 | 145.0 | 85.7 | 50 | 300 |  |  |  |
|  | W3 | 20 | 129.5 | 91.7 | 50 | 350 |  |  |  |
|  | W4 | 9 | 147.8 | 64.8 | 70 | 250 |  |  |  |
|  | Total | 42 | 141.7 | 82.5 | 50 | 350 | 2.511 | 3 | 0.473 |
| Orientation | W1 | 5 | 1.6 | 1.2 | 0 | 3.1 |  |  |  |
|  | W2 | 8 | 2.5 | 2.4 | 0 | 5.5 |  |  |  |
|  | W3 | 20 | 3.1 | 2.0 | 0 | 5.5 |  |  |  |
|  | W4 | 9 | 3.3 | 1.8 | 0 | 5.5 |  |  |  |
|  | Total | 42 | 2.8 | 2.0 | 0 | 5.5 | 2.978 | 3 | 0.395 |
| Distance to river | W1 | 5 | 92.0 | 87.0 | 0 | 200 |  |  |  |
|  | W2 | 8 | 40.6 | 50.6 | 0 | 150 |  |  |  |
|  | W3 | 20 | 118.5 | 340.6 | 0 | 1500 |  |  |  |
|  | W4 | 9 | 83.0 | 158.1 | 0 | 500 |  |  |  |
|  | Total | 42 | 92.9 | 246.3 | 0 | 1500 | 1.491 | 3 | 0.684 |

**Supplementary Figures**


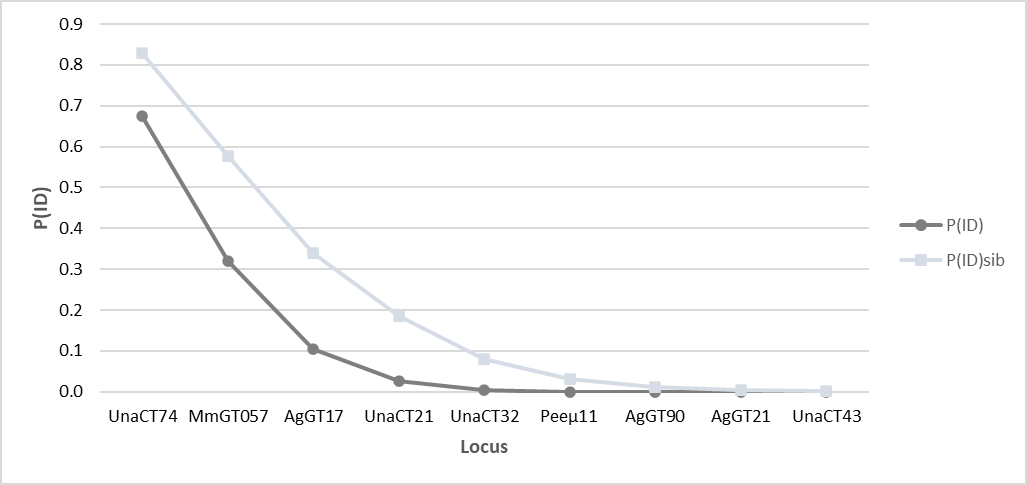


**Fig. S1.** Cumulative probabilities of identity (*P*_(ID)_ and *P*_(ID)sib_) for microsatellite loci in the red-fronted macaw.


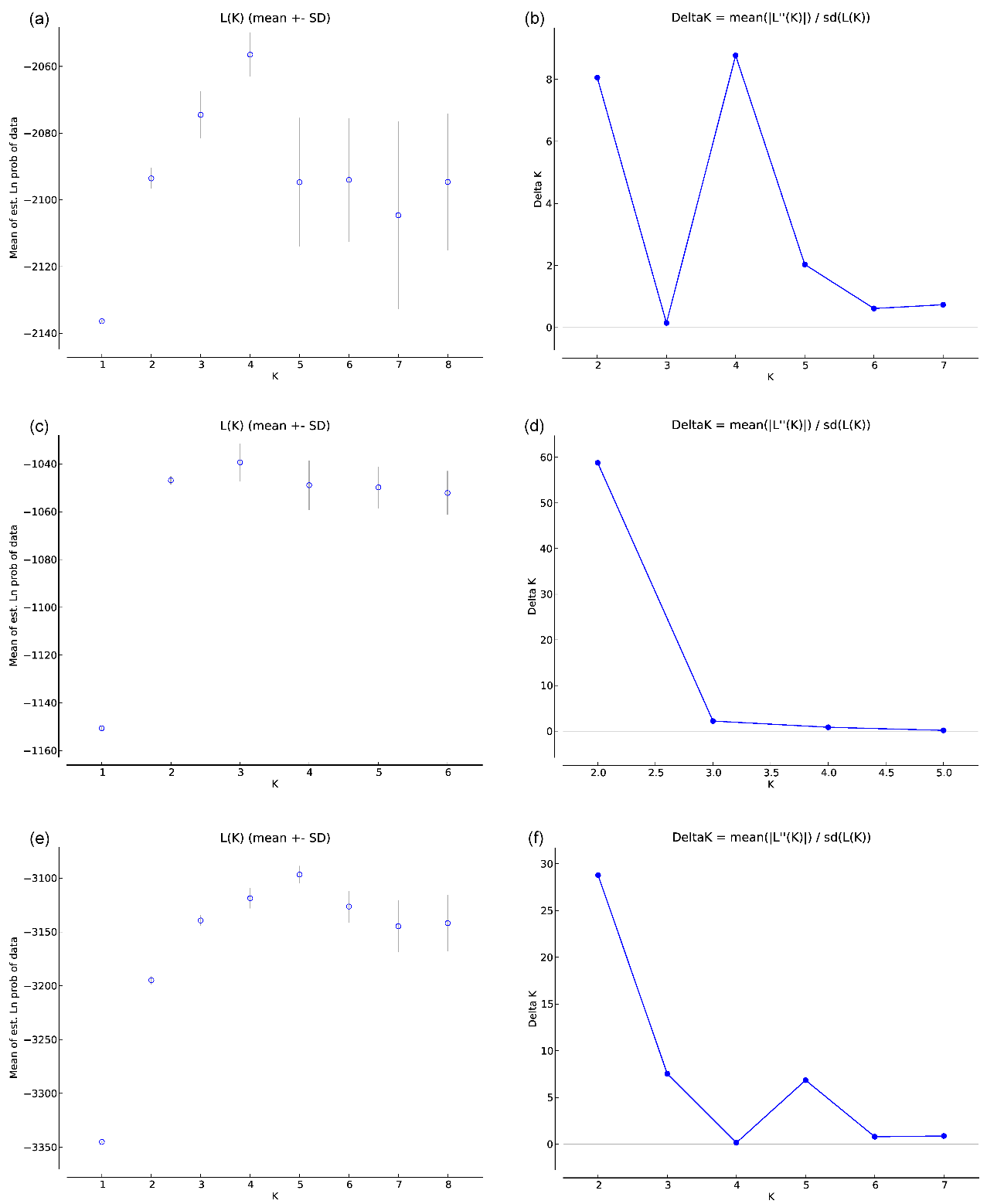


**Fig. S2.** Log-likelihood probability (LnP(*K*)) and *∆K* of the number of inferred clusters (*K*) in wild (a, b), captive (c, d), and combining wild and captive (e, f) of red-fronted macaw of different geographic areas.

**
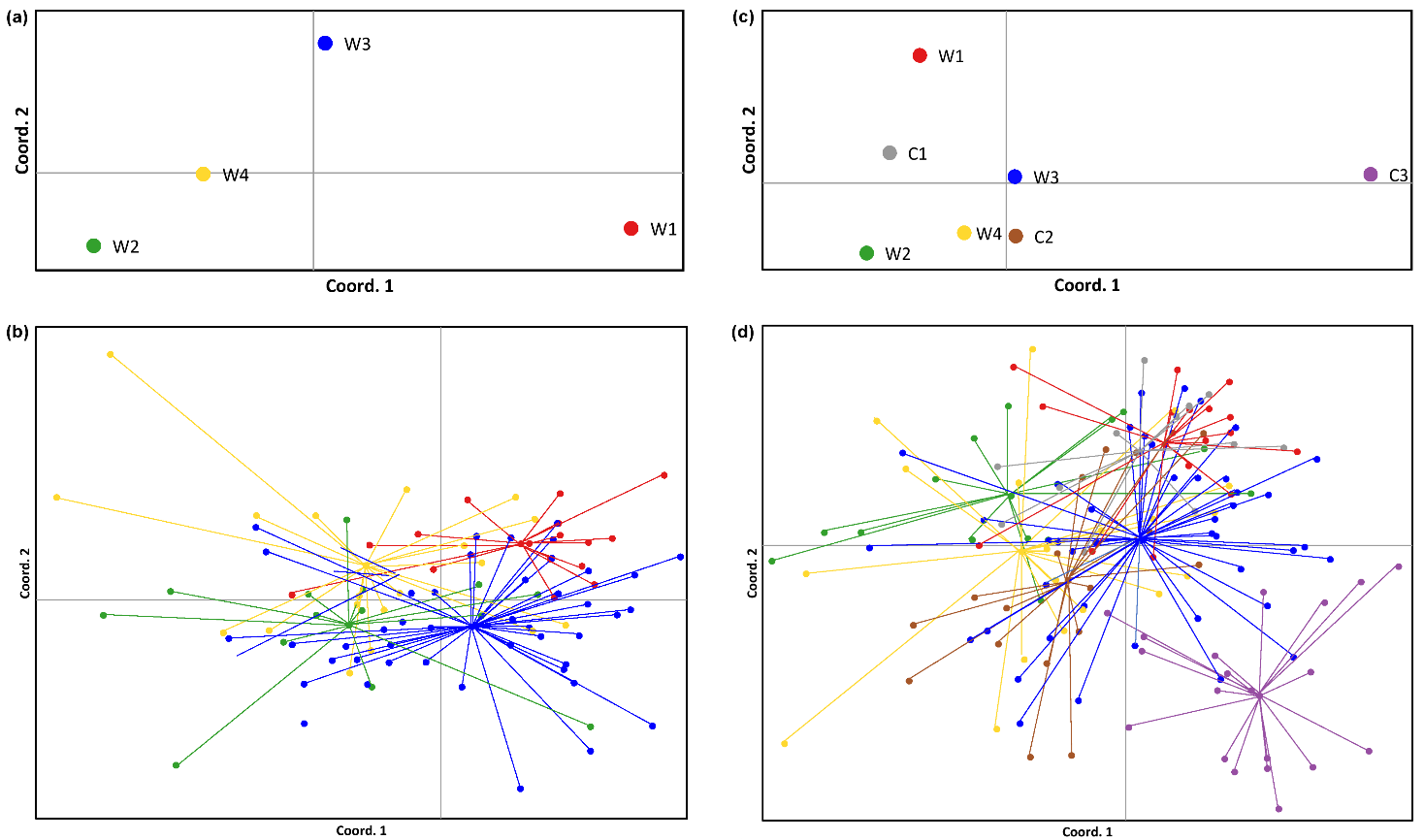
**

**Fig. S3.** PCoA plot of population (a, b) and individual (b, d) genetic distances calculated using the method of Smouse and Peakall (1999)^18^. (a) Between wild population groups (the first and second axes explain 64% and 20% of genetic variation, respectively); (b) between wild individuals (the first and second axes explain 14% and 10% of genetic variation, respectively); (c) between wild population groups and captive groups (the first and second axes explain 47% and 26% of genetic variation, respectively); and (d) between wild and captive individuals (the first and second axes explain 13% and 10% of genetic variation, respectively). Names of genetic clusters correspond to those shown in Table 2.


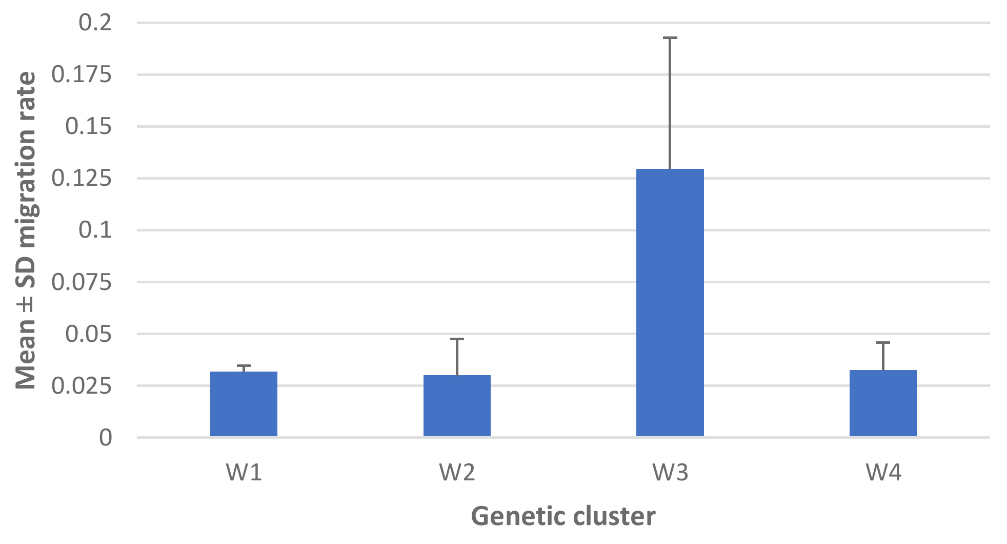


**Fig. S4.** Mean values of the migration rates *(m*), representing the proportions of immigrants estimated for each genetic cluster (W1-W4) of red-fronted macaw in the wild.


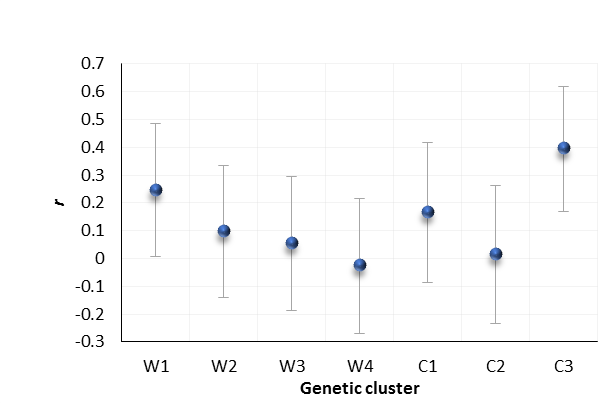


**Fig. S5.** Mean within-population pairwise values of relatedness values (*r*), according to Queller and Goodnight, (1989), in each genetic cluster of wild and captive red-fronted macaws. Error bars represent standard deviation (SD).

**Fig. S6.** Isolation by distance pattern among genetic clusters of wild red-fronted macaws considering (a) all population nuclei and (b) excluding the southern more isolated nuclei (W1).

**References for Supplementary Information**

1. Sambrook, J., Fritsch, E. & Maniatis, T. *Molecular Cloning: a Laboratory Manual* (Cold Spring Harbor Laboratory Press, New York, 1989).

2. Bello, N., Francino, O. & Sanchez, A. Isolation of genomic DNA from feathers. *J. Vet. Diagn. Invest.* **13**, 162–164; https://doi.org/10.1177/104063870101300212 (2001).

3. Russello, M., Calcagnotto, D., DeSalle, R. & Amato, G. Characterization of microsatellite loci in the endangered St. Vicent parrot, Amazona guildingii. *Mol. Ecol. Notes* **1**, 13–13; https://doi.org/10.1046/j.1471-8278.2001.00061.x (2001).

4. Taylor, T. D. & Parkin, D. T. Characterisation of 13 microsatellite loci for the Moluccan Cockatoo, *Cacatua moluccensis*, and Cuban Amazon, *Amazona leucocephala*, and their conservation and utility in other parrot species (Psittaciformes). *Conserv. Genet.* **8**, 991-994; https://doi.org/10.1007/s10592-006-9211-0 (2007).

5. Gebhardt, K. J. & Waits, L. P. Cross-species amplification and optimization of microsatellite markers for use in six Neotropical parrots. *Mol. Ecol. Resour.* **8**, 835-839; https://doi.org/10.1111/j.1755-0998.2007.02083.x (2008).

6. Russello, M. A., Saranathan, V., Buhrman‐Deever, S., Eberhard, J. & Caccone, A. Characterization of polymorphic microsatellite loci for the invasive monk parakeet (*Myiopsitta monachus*). *Mol. Ecol. Notes*, 7, 990-992; https://doi.org/10.1111/j.1471-8286.2007.01749.x (2007).

7. Valière, N. GIMLET: a computer program for analysing genetic individual identification data. *Mol. Ecol. Notes* **2**, 377-379; https://doi.org/10.1046/j.1471-8286.2002.00228.x-i2 (2002).

8. Belkhir, K., Borsa, P., Chikhi, L., Raufaste, N. & Bonhomme, F. GENETIX v. 4.05, Logiciel sous WindowsTM pour la Génétique des Populations. Laboratoire Génome et Population, Université Montpellier II, Montpellier, France (2004).

9. Bantock, T. M., Prys-Jones, R. P. & Lee, P. L. New and improved molecular sexing methods for museum bird specimens. *Mol. Ecol. Resour.***8**, 519-528; https://doi.org/10.1111/j.1471-8286.2007.01999.x (2008).

10. Griffiths, R., Double, M. C., Orr, K. & Dawson, R. J. A DNA test to sex most birds. *Mol. Ecol.* **7**, 1071-1075; https://doi.org/10.1046/j.1365-294x.1998.00389.x (1998).

11. Cornuet, J. M. & Luikart, G. Description and power analysis of two tests for detecting recent population bottlenecks from allele frequency data. *Genetics* **144**, 2001-2014 (1996).

12. Luikart, G., Allendorf, F. W., Cornuet, J. M. & Sherwin, W. B. Distortion of allele frequency distributions provides a test for recent population bottlenecks. *J. Hered.* **89**, 238-247; https://doi.org/10.1093/jhered/89.3.238 (1998).

13. Spencer, C. C., Neigel, J. E. & Leberg, P. L. Experimental evaluation of the usefulness of microsatellite DNA for detecting demographic bottlenecks. *Mol. Ecol.* **9**, 1517-1528; https://doi.org/10.1046/j.1365-294x.2000.01031.x (2000).

14. Garza, J. C. & Williamson, E. G. Detection of reduction in population size using data from microsatellite loci. *Mol. Ecol.* **10**, 305-318; https://doi.org/10.1046/j.1365-294X.2001.01190.x (2001).

15. Peery, M. Z. et al. Reliability of genetic bottleneck tests for detecting recent population declines. *Mol. Ecol.* **21**, 3403-3418; https://doi.org/10.1111/j.1365-294X.2012.05635.x (2012).

16. Wilson, G. A. & Rannala, B. Bayesian inference of recent migration rates using multilocus genotypes. *Genetics* **163**, 1177-1191 (2003).

17. Paetkau, D., Slade, R., Burden, M. & Estoup, A. Genetic assignment methods for the direct, real-time estimation of migration rate: a simulation‐based exploration of accuracy and power. *Mol. Ecol.* **13**, 55-65; https://doi.org/10.1046/j.1365-294X.2004.02008.x (2004).

18. Smouse, P. E., & Peakall, R. Spatial autocorrelation analysis of individual multiallele and multilocus genetic structure. *Heredity* **82**, 561-573; https://doi.org/10.1038/sj.hdy.6885180 (1999).
